# A Voucher Flora of Diatoms from Fens in the Tanana River Floodplain, Alaska

**DOI:** 10.3390/w15152803

**Published:** 2023-08-02

**Authors:** Veronica A. Hamilton, Sylvia S. Lee, Allison R. Rober, Paula C. Furey, Kalina M. Manoylov, Kevin H. Wyatt

**Affiliations:** 1 Department of Biology, Ball State University, Muncie, IN 47306, USA; 2 Office of Research and Development, U.S. Environmental Protection Agency, Washington, DC 20460, USA; 3 Department of Biology, St. Catherine University, St. Paul, MN 55105, USA; 4 Department of Biological & Environmental Sciences, Georgia College & State University, Milledgeville, GA 31061, USA

**Keywords:** algae, Bacillariophyceae, biofilm, climate change, freshwater, microalgal diversity, periphyton, voucher specimens

## Abstract

Climate change and human activities may alter the structure and function of boreal peatlands by warming waters and changing their hydrology. Diatoms can be used to assess or track these changes. However, effective biomonitoring requires consistent, reliable identification. To address this need, this study developed a diatom voucher flora of species found across a boreal fen gradient (e.g., vegetation) in interior Alaskan peatlands. Composite diatom samples were collected bi-weekly from three peatland complexes over the 2017 summer. The morphological range of each taxon was imaged. The fens contained 184 taxa across 38 genera. *Eunotia* (45), *Gomphonema* (23), and *Pinnularia* (20) commonly occurred in each peatland. *Tabellaria* was common in the rich and moderate fen but sparse in the poor fen. *Eunotia* showed the opposite trend. Approximately 11% of species are potentially novel and 25% percent matched those at risk or declining in status on the diatom Red List (developed in Germany), highlighting the conservation value of boreal wetlands. This voucher flora expands knowledge of regional diatom biodiversity and provides updated, verifiable taxonomic information for inland Alaskan diatoms, building on Foged’s 1981 treatment. This flora strengthens the potential to effectively track changes in boreal waterways sensitive to climate change and anthropogenic stressors.

## 1. Introduction

A number of diatom (Bacillariophyceae) taxa respond quickly to environmental change and have been used as effective indicators of climate in the circumpolar Arctic [1]. Their specific range preferences, along with the morphologically distinct features of their frustules, allow for taxonomic differentiation to the species level. The durability of their silica cell wall is valuable for the examination of present as well as past environments [2,3]. However, to successfully investigate diatom ecology, determine patterns in biogeography, and use species identity as indicators of environmental condition, their identification must be as unambiguous as possible and verifiable (i.e., documented with images and traceable back to archived material and references) [4]. The lack of complete and accessible taxonomic guides for species-level identification makes this particularly challenging [5].

An accepted technique for verifying taxonomic identity in biological surveys is to create and maintain permanent archives of voucher specimens for use as reference guides [6]. For diatoms, permanent microscope slides are deposited in public herbaria and maintained for federal and state programs at entities such as the Diatom Herbarium at the Academy of Natural Sciences of Drexel University (ANS—Philadelphia, Pennsylvania, USA) or other museum collections. However, the labor-demanding and cost-prohibitive efforts to document individual specimens often prevent research teams from comprehensively designating representatives on slides (via circling individual diatom specimens) of all observed taxa in their samples [7]. When made accessible, documentation of project-specific morphological species boundaries (through digital images) can aid in taxonomic harmonization with current and future monitoring data, maximize data use, and maintain informative long-term records [8].

A voucher flora is a document that records specimens through images and their nomenclatural designations. It creates a visual record for a given project. Taxonomic voucher floras are tied to specimens made publicly available in herbaria. They help align taxonomists’ morphological species concepts during analysis in large studies involving multiple taxonomists. This allows for taxonomic verification of specimens through representative images, grants reinterpretation of names applied to specimens in future investigations, and facilitates taxonomic continuity in identification over time, especially in long-term ecological studies [9,10]. Voucher floras are collaborative documents that facilitate taxonomic discussions and interpretations within and between labs. Long-term diatom studies often require multiple taxonomists; voucher floras provide complete documentation for taxonomists to overcome hurdles to directly communicate with each other to align their concepts of morphological species boundaries used in a project. Without documentation of taxa in a voucher flora, extensive post hoc harmonization may be required to reduce data errors, usually at the cost of losing species information [11,12]. Voucher floras provide a series of digital images that document the full morphological range of voucher specimens as well as complete reference information. This is not only more information than a list of taxa, but is also more practical than a set of circled specimens (though, when available, archives of permanent slides remain important resources) [9]. Given the expeditious development of freshwater diatom taxonomy, coupled with high degrees of endemism and species diversity, no single taxonomic reference adequately supports species-level identification of all taxa in a given project [13]. Thus, developing taxonomic reference voucher floras for localized regions becomes vital for supporting long-term records, promoting efficient verification of species richness and the assessment of diatom assemblage structure.

Information on diatom taxonomy is not ubiquitous across all areas, with some regions better represented in the literature than others. Floristic studies of freshwater diatom taxa in North America remain sparse relative to the size and diversity of habitats [14] compared to floristic studies of Europe, for example. Boreal regions in North America are especially underrepresented despite the amount of open water areas present at northern latitudes [15]. In Alaska alone, open water environments comprise more than half of the state’s total surface area [15]. Thus, an increased demand for regional voucher floras is emerging as taxonomists attempt to harmonize identification across broader spatial scales [4,9,11]. Diatomists often rely on taxonomic information from European references despite this information being applied to European waters. This further highlights the need for region-specific floras in other areas of the world, especially with the growing descriptions of species new to science [16–18], documentation of endemic taxa [19–21], and establishment of new species records [22,23] within North America. Furthermore, there is no diatom “Red List” of threatened species currently available for Alaska or the United States as a whole; thus, referencing the diatom Red List developed in Germany [24,25] can help further the conversation around imperiled diatom taxa and the urgent need to conserve their habitats.

Approximately 85% of the open water areas of Alaska are classified as wetlands [15]. Peatlands are a common type of wetland habitat in Alaska. Peat forms and accumulates through a complex biogeochemical process, driven by the slow decomposition of dead plant matter due to cold, nutrient-poor, anaerobic conditions related to water saturation [26]. Traditionally, diagnostic tools based on plants distinguish wetland types in Alaska where sharp vegetative boundaries between bogs and fens emerge from contrasting hydrologic properties [27]. Recent studies in boreal peatlands reveal that diatoms and other microalgae can be abundant [28–30] and can regulate many aspects of biogeochemical cycling [31,32]. Floras of microalgae complement these ecological studies, especially to inform future studies in boreal peatlands about biological changes in response to climate change and other anthropogenic stressors.

This study aims to document the species richness of diatom assemblages across a gradient of boreal peatlands to build an image-rich voucher flora for use as a diagnostic tool in future studies. We investigated diatom species composition in three peatland complexes just outside the Bonanza Creek Experimental Forest in interior Alaska. We expected to find diatom assemblages containing characteristic minerotrophic, acidophilous, and epiphytic taxa, based on recent studies in other high-latitude wetlands [33]. We aimed to capture the full morphological size range of each species encountered, known as their operational taxonomic unit (OTU), when arranged on voucher plates [11]. This size diminution series documents how species’ morphological characteristics change across their life cycle, providing valuable information about the morphological variation expected during identification and enumeration. This localized voucher flora of boreal peatlands in interior Alaska is hereafter referred to as the Alaskan Peatland Project (APP). It answers the recent call to action for more region-specific diatom floras and aligns with modern taxonomic efforts to communicate taxonomic practice and to provide accessible identification resources for taxonomic consistency at federal, state, and local levels [4]. This study provides a focused, image-rich look at diatom species assemblages in an area of the world that is changing owing to anthropogenic activities.

## 2. Materials and Methods

### 2.1. Study Sites

This study was conducted in three peatlands (a rich, moderate, and poor fen) located within a wetland complex in the Tanana River floodplain just outside the 12,486-acre Bonanza Creek Experimental Forest (35 km southeast of Fairbanks) in interior Alaska, USA (64°42′ N, 148°18′ W). This area is part of the circumpolar range of boreal forest, with the Tanana River valley positioned 150–250 km south of the Arctic Circle. Minerotrophic peatlands with distinctive vegetation communities and water chemistry are referred to as fens [34]. Rich fens, the most common boreal peatland type in North America [34], have a pH that ranges from 6.8–8 and high concentrations of dissolved minerals to support a diversity of vegetation types, including sedges, shrubs, and brown mosses. Moderate fens have a pH range of 5–7 and are moderately rich in dissolved minerals and vegetation diversity, including sedges and brown mosses with sparsely distributed *Sphagnum* moss. Poor fens, with a pH range of 4–5.5 and low concentrations of dissolved minerals, are dominated by *Sphagnum* moss, a species capable of acidifying the surrounding environment and thereby inhibiting many vascular plants [34].

Each fen site selected for this study was classified prior to this study using natural transitions in vegetation community structure and water chemistry [35,36]. A full description of fen characteristics is presented in Ferguson et al. [30], but briefly, the rich fen was approximately 200 m^2^ in size and comprised of brown moss species (families Amblystegiaceae and Brachytheciaceae) and emergent vascular plants (*Carex atherodes*, *Equisetum fluviatile*, and *Potentilla palustris*). The moderate fen was approximately 100 m^2^ in size and contained both brown moss and *Sphagnum* species with vegetation comprised of *C. atherodes*, *E. fluviatile*, and *P. palustris*. The poor fen was 30 m^2^ in size and was primarily composed of *Sphagnum* species with *E. fluviatile*, *P. palustris*, and *Eriophorum vaginatum*. The fens in our study were not directly connected but were located within ~1 km distance from one another. Each fen site was completely saturated with standing water for the entirety of the growing season [30], which is reflected in fen physical and chemical characteristics present at the time of sampling (Table 1).

### 2.2. Experimental Design and Sample Processing

Diatom samples were collected during the growing season of 2017 (29 May–1 August 2017) from each of the three fen sites every 10–14 days at four locations (1 m^2^ plots). The one-meter-squared plots each consisted of four 25 cm^2^ areas. Samples from each of the four areas were composited into a single vial. Each sample consisted of loosely attached algae and periphyton collected with a syringe from the peat surface (when present), and the submersed portions of four stems of the dominant emergent macrophyte were scraped with a toothbrush then combined to form a total of 72 composite samples (24 per fen). The samples were preserved in a 2% formalin solution, transported back to the laboratory, and stored for processing and analysis.

Prior to identification, samples for diatom identification were acid-cleaned by adding hydrochloric acid and boiling to remove organic matter from within the diatom valves and rinsing the samples with distilled water until the acid was neutralized [7]. Cleaned, concentrated siliceous material was then dripped onto three separate 18 × 18 mm coverslips per sample and allowed to air dry. Each coverslip was visually inspected for the appropriate density of cells (15–30 visible valves per field of view at 400× magnification following NAWQA protocol) prior to permanent fixation to microscope slides with Naphrax^™^ (Brunel Microscopes Ltd., Chippenham, UK) mounting medium [7]. All slides were visually scanned transect after transect to completion (to include each of the triplicate slides for each sample) with adjustments to see entire specimens (if part of it was in the transect) and digitally photomicrographed using a 100× oil immersion objective on a Leica DM6B light microscope with 19-mm sCMOS camera (Leica Microsystems, Wetzlar, Germany). Diatom measurements (length, width, and stria density) were taken with ImageJ 1.53e (NIH, Bethesda, MD, USA) software [37] and diatom size diminution series were organized into morphological operational taxonomic units (OTU). No valve counts or enumeration were conducted during the construction of the voucher flora. Efforts were made to image all suitable valves encountered for future research.

Initial species identification and nomenclature followed Kramer and Lange-Bertalot [38–41], Patrick and Reimer [42,43], Krammer [44], Lange-Bertalot and Kramer [45], Lange-Bertalot et al. [46], Lange-Bertalot et al. [47], and Diatoms of North America [4]. Literature specific to Western North America and Alaska: Bahls [16], Bahls et al. [48], Bahls and Luna, [49] and Foged [50] allowed for critical evaluation of taxonomy and refinement of species complexes to sensu stricto taxa (see Supplementary Materials, File S1: taxonomic authority references). Images for publication of the regional voucher flora were produced using Leica LAS X 5.1.0 imaging software and Adobe Photoshop v 24.7. Images of specimens were imported as layers and arranged by OTU onto plates but were not manipulated or altered in Photoshop.

### 2.3. Data Analysis

We calculated similarity in assemblage composition between all site pair combinations (e.g., rich fen vs. moderate fen) using the Sørensen coefficient on presence–absence data (see Supplementary Material, Table S1: species information). This coefficient ranges from 0 to 1, with high values indicating closely similar species composition between two sites. A recent study comparing the performance of several similarity indices recommended the use of the Sørensen index for analyzing binary community data [51], common in community ecology.

## 3. Results

A total of 184 taxa from 38 genera were identified to the lowest taxonomic level possible (Table 2; Plates 1–25). Of the diatom taxa, 129 were formally described species known in the published literature, 34 were listed with confer/conferatur (cf.) and 21 were presented at genus level with a provisional name assigned for this project (e.g., *Tabellaria* sp.1 APP). Some of the more infrequent species remain undescribed but contain size ranges (Table 2) and images (see plates). Approximately 11% of the documented species across all fens were potentially new to science; therefore, a provisional name, over a formal name, was assigned to assist in future enumeration efforts.

In the Class Coscinodiscophyceae (4), the genera recorded comprised of *Aulacoseira, Lindavia, Melosira*, and *Stephanocyclus*. In the Class Fragilariophyceae (5), representatives of *Diatoma, Fragilaria, Staurosira, Staurosirella*, and *Tabellaria* were documented. The highest species richness was recorded in the Class Bacillariophyceae (26), (which is to be expected for substrate-attached benthic habitats) and included the monoraphid, asymmetric biraphid, symmetric biraphid, nitzschioid, surirelloid, and eunotioid taxa. Within this Class, the most speciose genera were *Eunotia, Gomphonema, Pinnularia, Navicula*, and *Nitzschia*.

### 3.1. Distribution of Common Taxa

**Plate 2.**
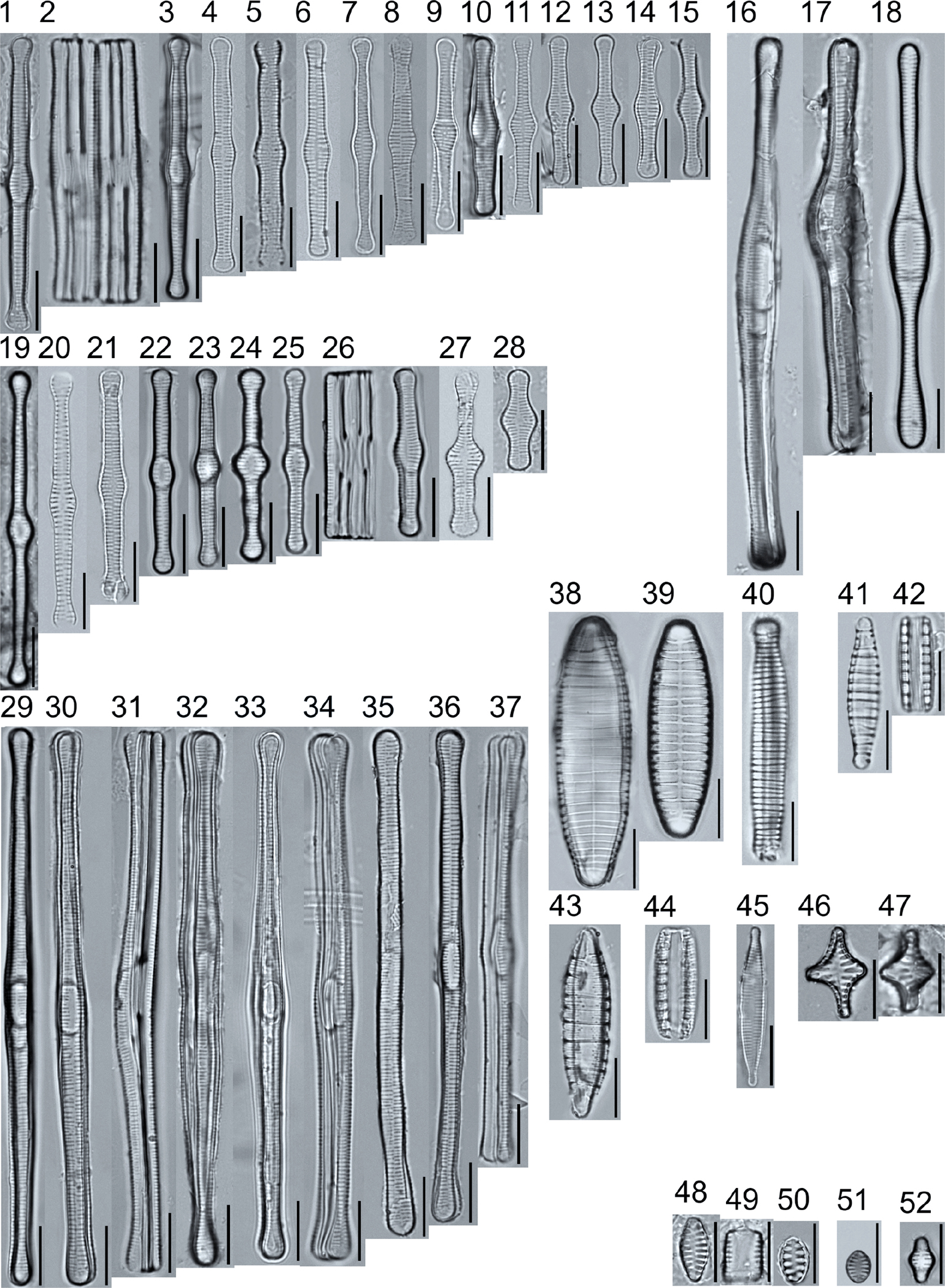
Light micrographs of araphid taxa. Figs. 1–15: *Tabellaria fenestrata*. Figs. 16–18: *T*. sp.1 APP. Figs. 19–28: *T. flocculosa*. Figs. 29–37: *T*. sp.2 APP. Figs. 38–39: *Diatoma vulgaris*. Figure 40: *D. ehrenbergii*. Figs. 41–42: *D. moniliformis*. Figure 43: *Odontidium hyemale*. Figure 44, Girdle view: *Denticula* cf. *kuetzingii?* Figure 45: *Fragilaria rumpens*. Figs. 46–47: *Staurosirella leptostauron*. Figs. 48–50: *S. pinnata*. Figure 51: *Staurosira construens* var. *venter*. Figure 52: *Staurosira* cf. *construens*. Scale bars: 10 μm.

**Plate 5.**
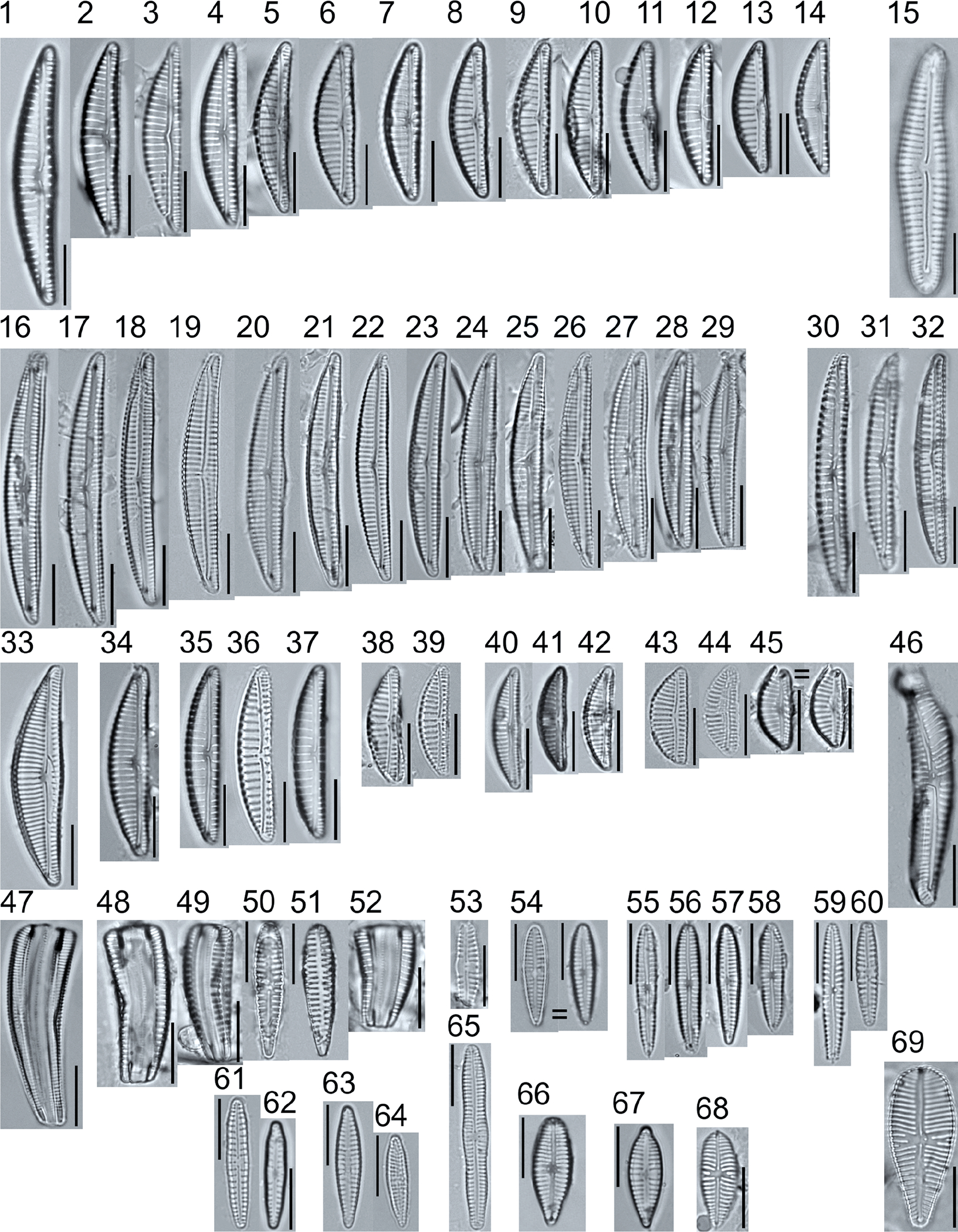
Light micrographs of asymmetric biraphid taxa. Figs. 1–14: *Encyonema paucistriatum*. Figure 15: *E*. sp.1 APP. Figs. 16–29: *E. neogracile*. Figs. 30–32: *E. lunatum* var. *alaskaense*. Figs. 33: *E. silesiacum*. Figure 34: *E. procerum*. Figs. 35–37: *E. groenlanica*. Figs. 38–39: *E*. cf. *groenlandica*. Figs. 40–42: *E. schimanskii*. Figs. 43–45: *E. montana*. Figure 46: *E*. sp.2 APP (Asymmetric teratological form). Figure 47: *Rhoicosphenia* cf. *stoermeri*. Figs. 48–52: *R. abbreviata*. Figure 53: *Reimeria sinuata*. Figure 54: *Gomphonema* cf. *clavatulum*. Figs. 55–58: *G*. cf. *frigidum*. Figs. 59–60: *G*. cf. *parapygmaeum*. Figs. 61–62: *G. montanum* var. *minutum*. Figs. 63–64: *G*. cf. *consector*. Figure 65: *G. barrowiana*. Figure 66: *G. parvulum* f. *saprophilum*. Figure 67: G. cf. *himalayaense*. Fig. 68: *G. olivaceum* var. *densestriatum*. Figure 69: *G. italicum*. Scale bars: 10 μm.

**Plate 11.**
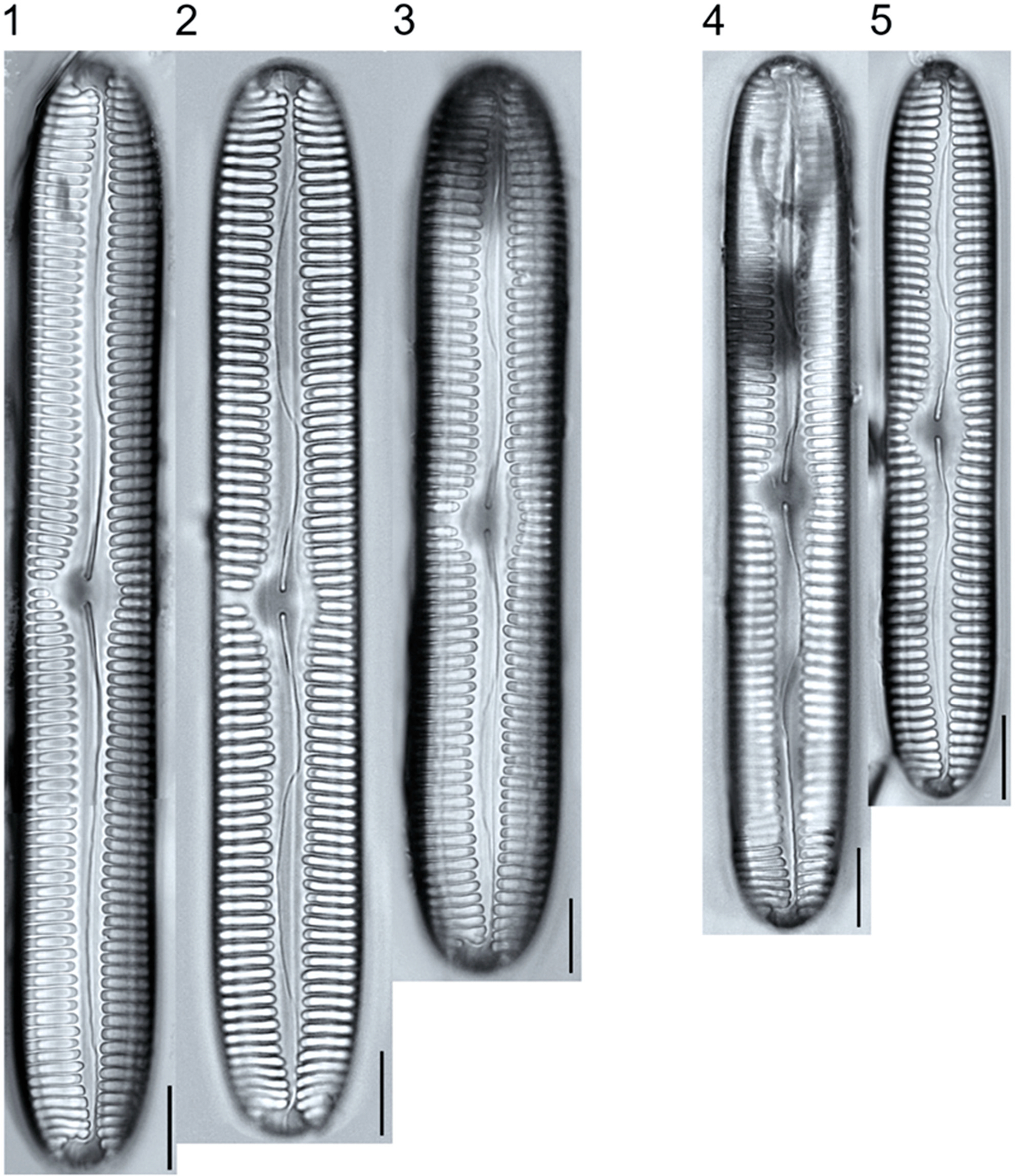
Light micrographs of symmetric biraphid taxa. Figs. 1–3: *Pinnularia genkalii*. Figs. 4–5: *P. ilkaschoenfelderae*. Scale bars: 10 μm.

*Tabellaria*, including undescribed species (*T*. sp.1 APP and *T*. sp.2 APP), was frequently encountered in the rich and moderate fens (Plate 2, Figs. 1–37). *Tabellaria flocculosa* (Roth) Kützing 1844 (p. 127) was encountered in each fen on every sampling date. Though *T. flocculosa* was the most common species in the rich fen, populations of *Eunotia pseudoflexuosa* Hustedt 1949 (p. 71) and *Tabellaria fenestrata* (Lyngbye) Kützing 1844 (p. 127) also frequently occurred. *Navicula* (pl. 8) and *Nitzschia* (pl. 17), both encountered infrequently in all fens, were more speciose where present, particularly within the rich fen. The rich fen also contained infrequent populations of *Cocconeis* Ehrenberg 1838 (p. 194), of which *C. pediculus* Ehrenberg 1838 (p. 194) was the most common. *Encyonema neogracile* Krammer 1997 (p. 177) and *E. paucistriatum* (Cleve-Euler) D.G. Mann 1990 (p. 667) were the most commonly encountered species of the nine representative species of *Encyonema* Kützing 1834 (p. 583) (Plate 5, Figs. 1–45). For a few of these taxa (Pl. 5, Figure 15. *E*. sp.1 APP; Pl. 5, Figure 46. *E*. sp.2 APP; Pl. 24, Figure 12. *Eunotia* sp.1 APP), teratological form is suspected owing to morphological abnormalities and infrequency of detection (discussed below).

**Plate 12.**
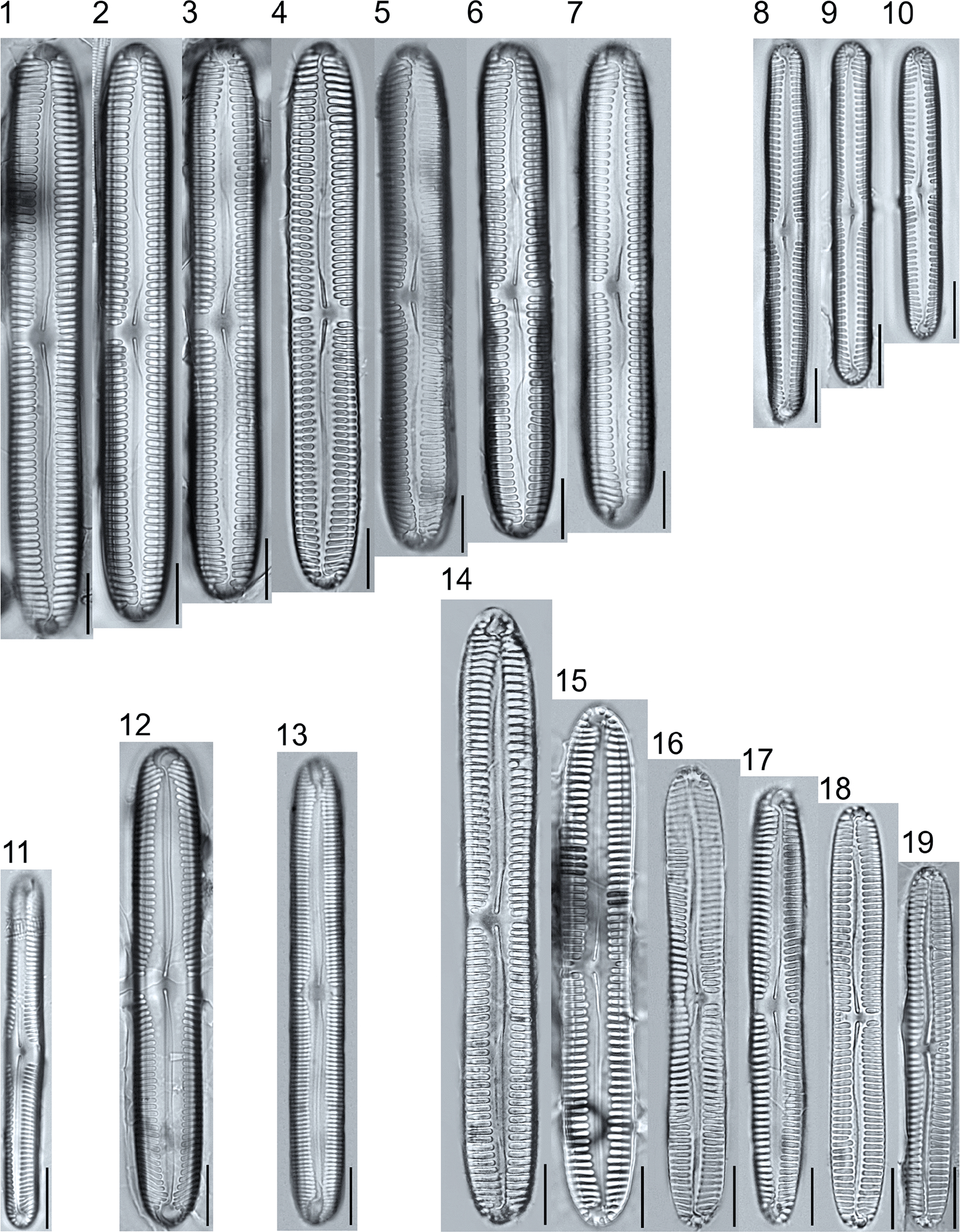
Light micrographs of symmetric biraphid taxa. Figs. 1–7: *Pinnularia moelderi*. Figs. 8–10: *P. abaujensis* var. *subundulata*. Figure 11: *P*. sp.1 APP. Figure 12: *P. cruxarea*. Figure 13: *P. spitsbergensis*. Figs. 14–19: *P*. sp.2 APP. Scale bars: 10 μm.

*Pinnularia pulchra* Østrup 1897 (p. 253), along with *Eunotia pseudoparallela* Cleve-Euler 1934 (p. 24), were common in the moderate fen. Of the 20 represented species of the genus *Pinnularia* (Plate 12, Figs. 14–20) a number remain undescribed (*P*. sp.1 APP and *P*. sp.2 APP). The moderate fen also supported 23 distinct OTUs of *Gomphonema*. Initial scans for the voucher flora frequently detected *Gomphonema hebridense* Gregory 1854 (p. 607), *Gomphonema brebissonii* Kützing 1849 (p. 66), and *Gomphonema* cf. *raraense* Jüttner and S. Gurung 2018 (p. 301) in the moderate and rich fens, but not in the poor fen. The genus *Stauroneis* Ehrenberg 1843 (p. 311) was rarely detected; however, the majority of species within the genus occurred in the moderate fen. Though centric taxa were infrequently observed during scanning, the genus *Lindavia* occurred in the greatest quantity. *Lindavia ocellata* (Pantocsek) Nakov et al. 2015 (p. 256) had the narrowest spatial and temporal distribution but was only detected in the moderate fen on one sampling date.

**Plate 19.**
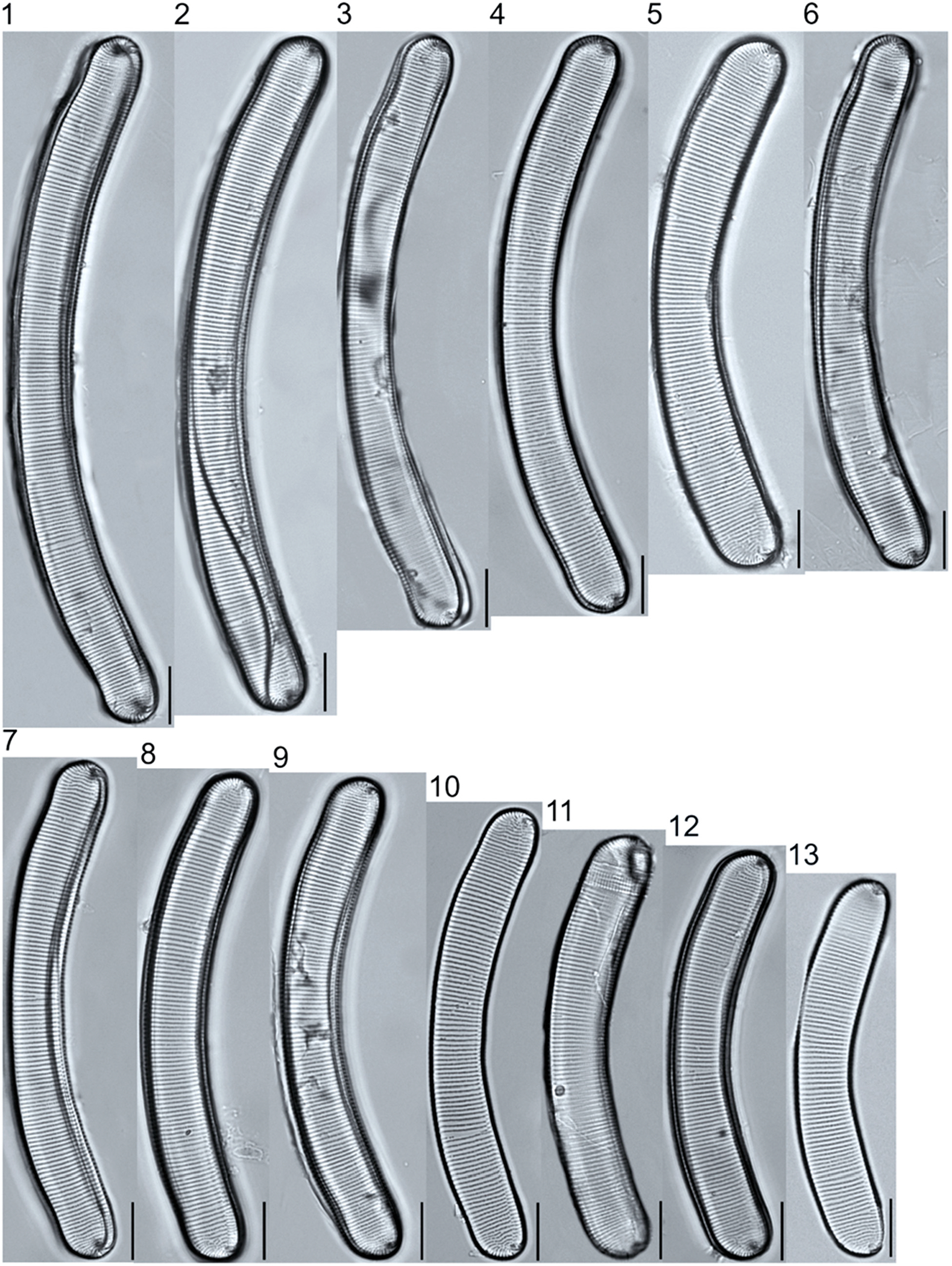
Light micrographs of eunotioid taxa. Figs. 1–13: *Eunotia pseudoparallela*. Scale bars: 10 μm.

**Plate 20.**
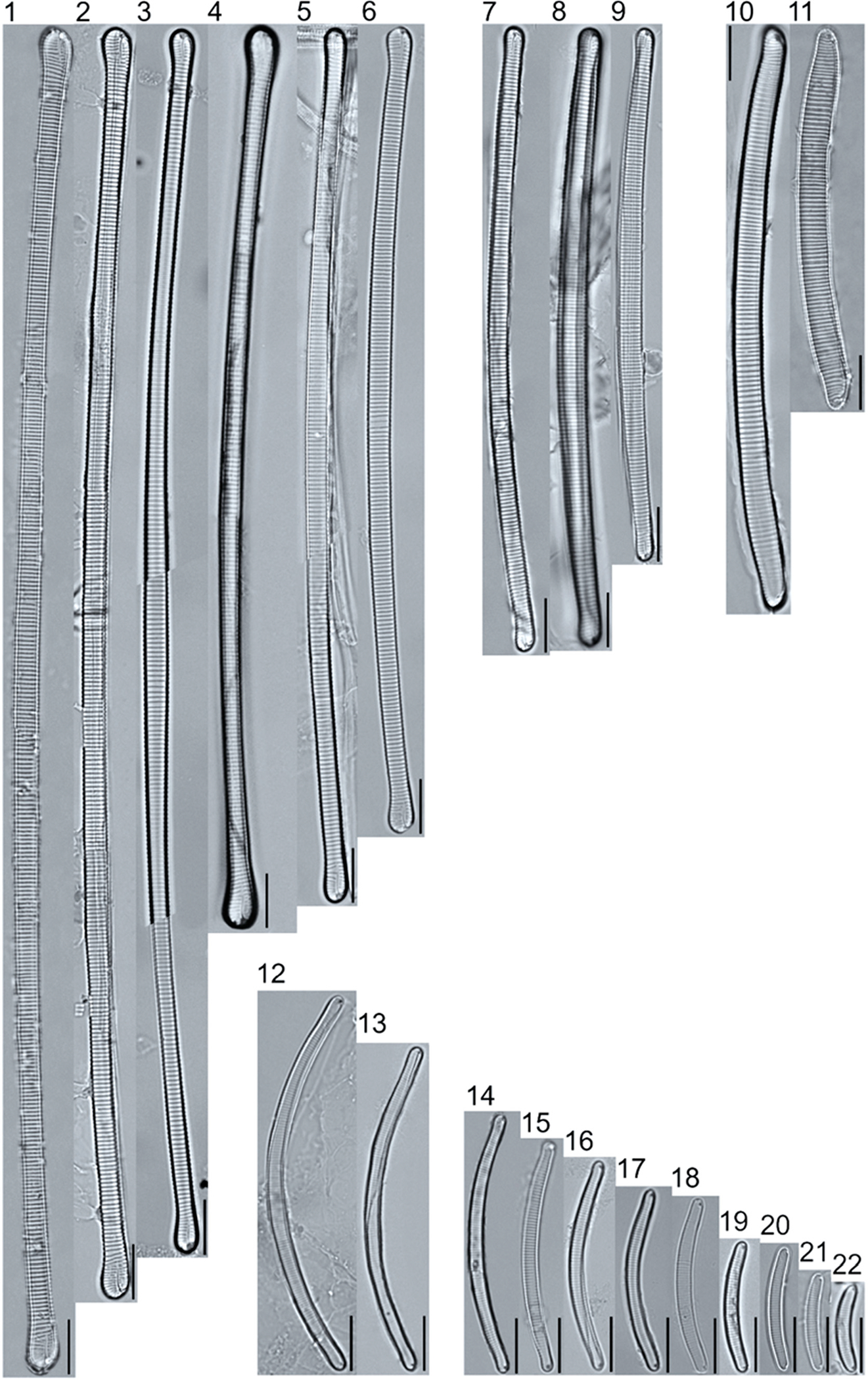
Light micrographs of eunotioid taxa. Figs. 1–6: *Eunotia flexuosa*. Figs. 7–9: *E. pseudoflexuosa*. Figs. 10–11: *E. valida*. Figs. 12–13: *E*. cf. *mucophila*. Figs. 14–22: *E. mucophila*. Scale bars: 10 μm.

**Plate 21.**
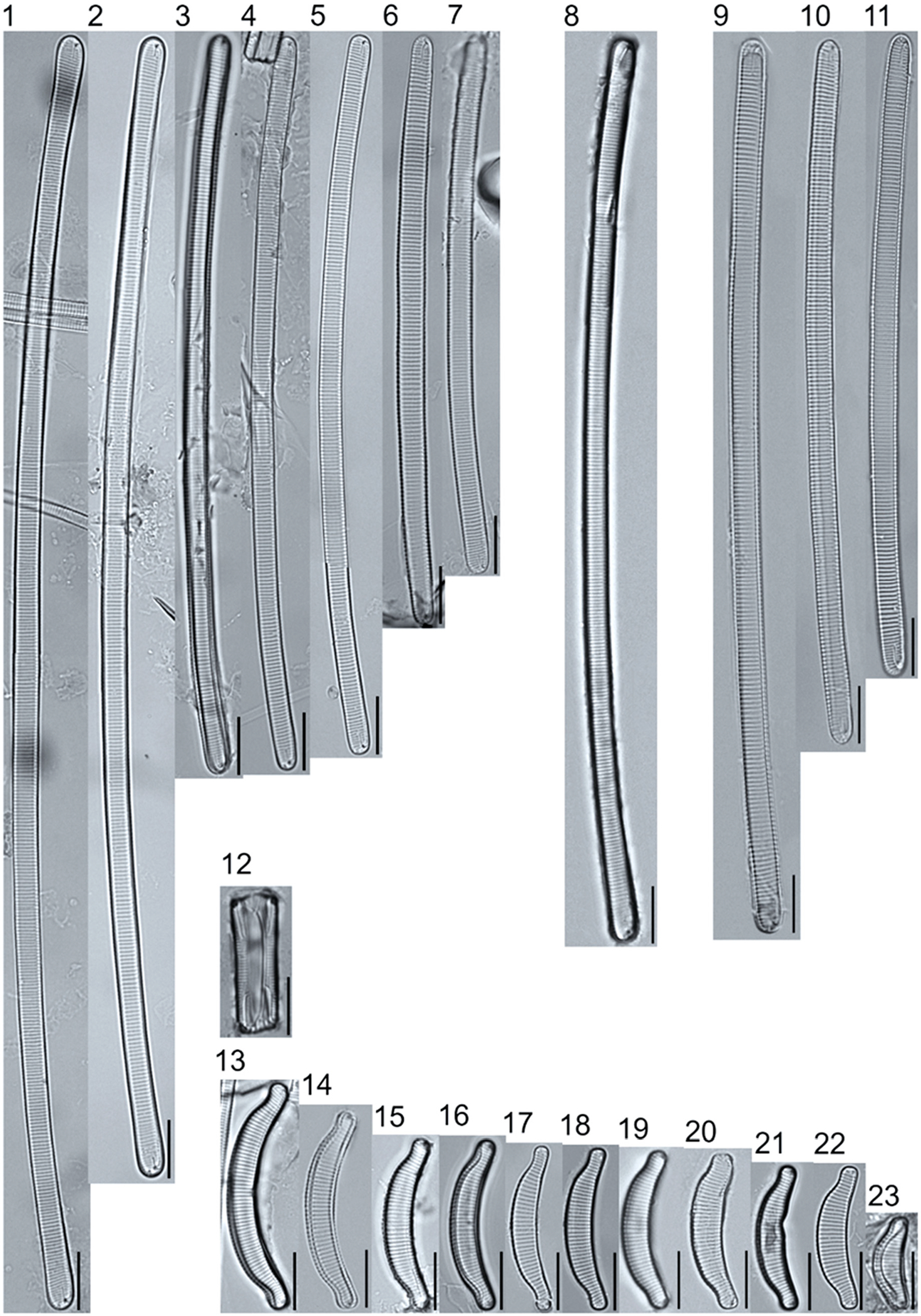
Light micrographs of eunotioid taxa. Figs. 1–7: *Eunotia julma* (Figs. 1–2: Apices may be slightly inflated at upper size limit—LB 2011, pg. 130), Figure 8: *E*. cf. *julma*. Figs. 9–11: *E. ambivalens*. Figs. 12–23: *E. ursamaioris*. Scale bars: 10 μm.

**Plate 22.**
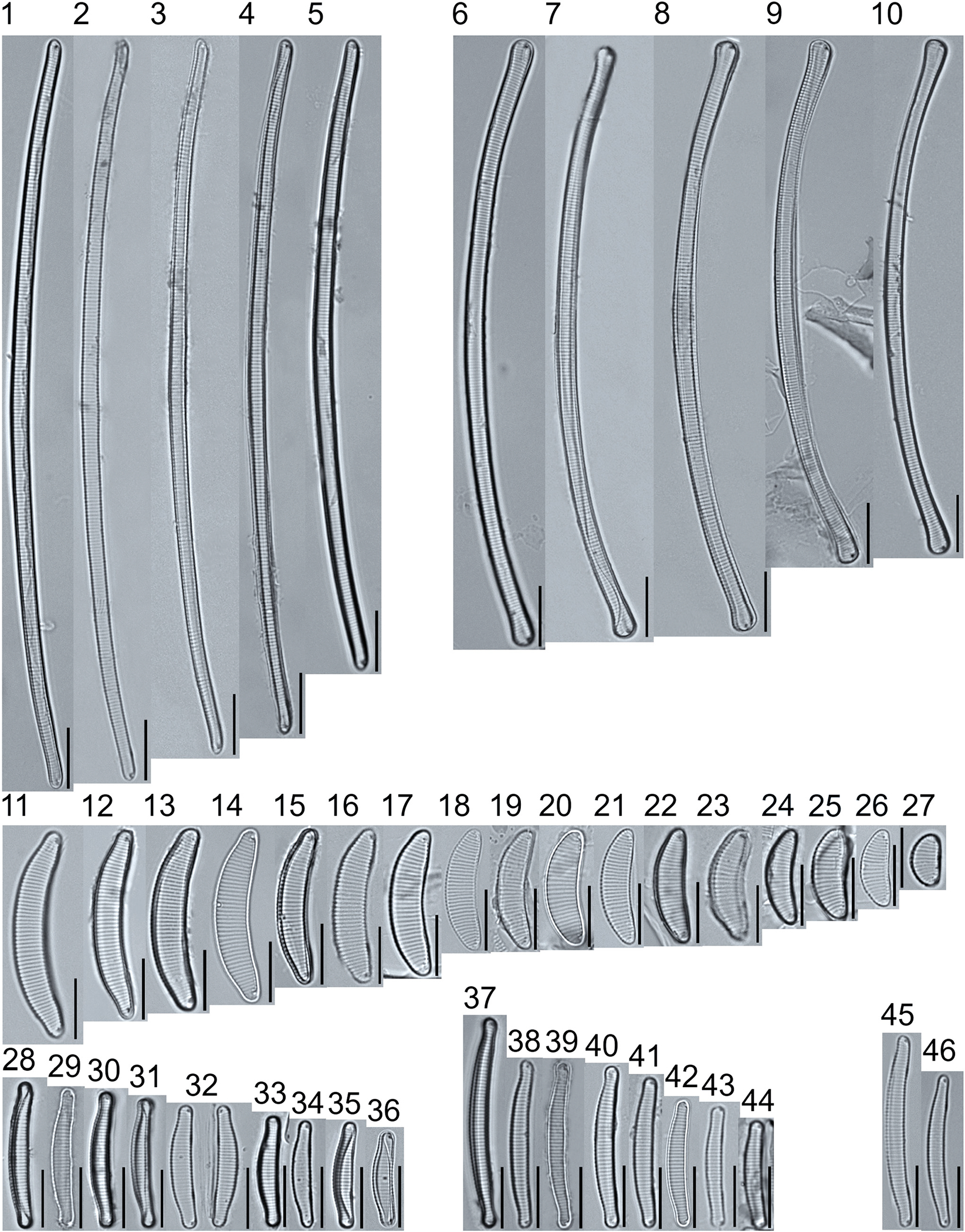
Light micrographs of eunotioid taxa. Figs. 1–5: *Eunotia naegelii*. Figs. 6–10: *E. subcapitata*. Figs. 11–27: *E. scandiorussica*. Figs. 28–36: *E*. cf. *bertrandii*. Figs. 37–44: *E. pseudogroenlandica*. Figs. 45– 46: *E*. cf. *paludosa* (compare with *E. pauldosa*; see Plate 25). Scale bars: 10 μm.

**Plate 23.**
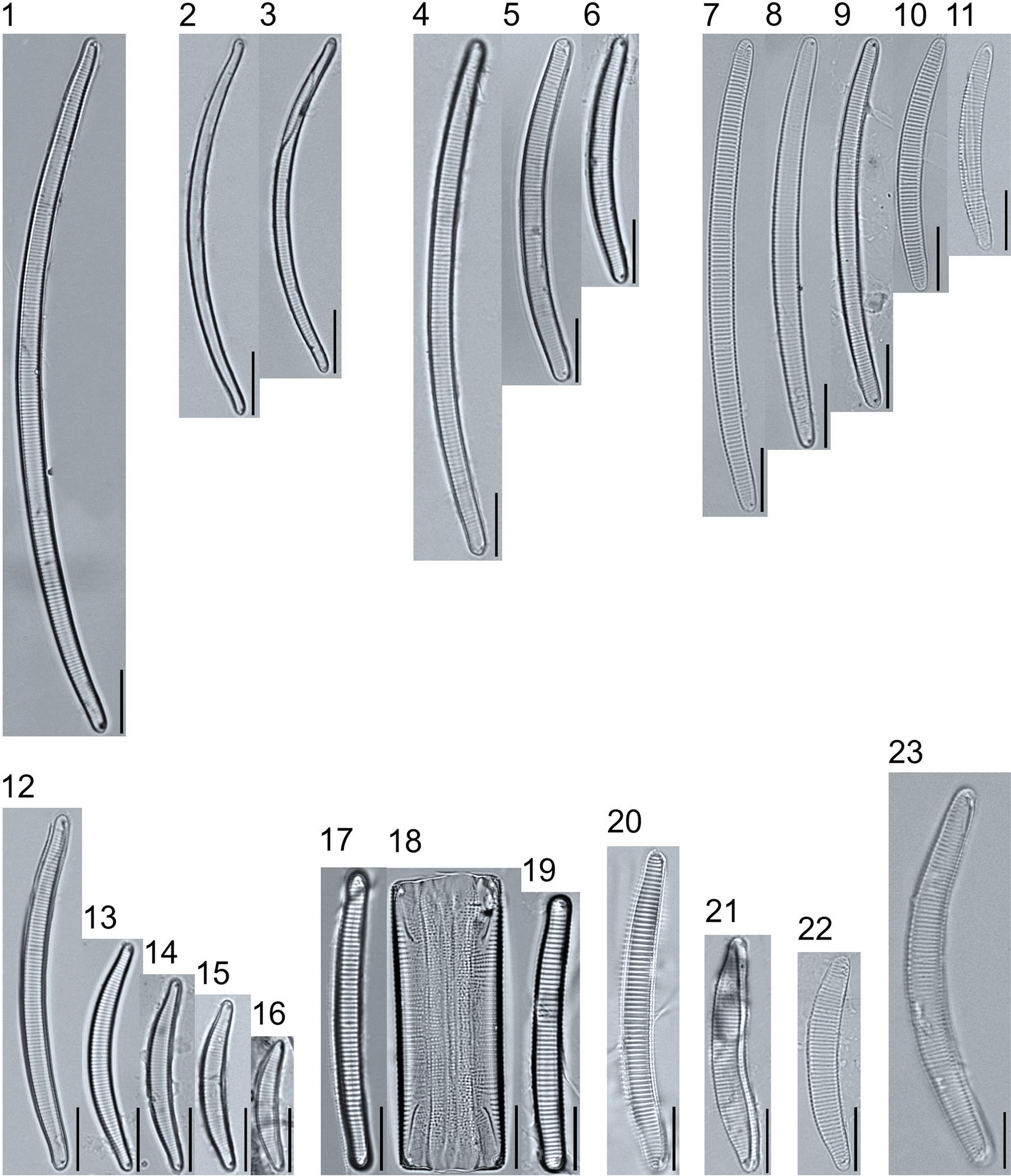
Light micrographs of eunotioid taxa. Fig 1: *Eunotia juettnerae*. Figs. 2–3: *E. krammeri*. Figs. 4–6: *E. panda*. Figs. 7–11: *E. bilunaris*. Figs. 12–16: *E. ferefalcata*. Figs. 17–19: *E*. cf. *glacialispinosa*. Figure 20: *E*. cf. *groenlandica*. Figure 21: *E*. cf. *silesioscandica*. Figure 22: *E*. cf. *scandiorussica*. Figure 23: *E*. sp.2 APP. Scale bars: 10 μm.

**Plate 24.**
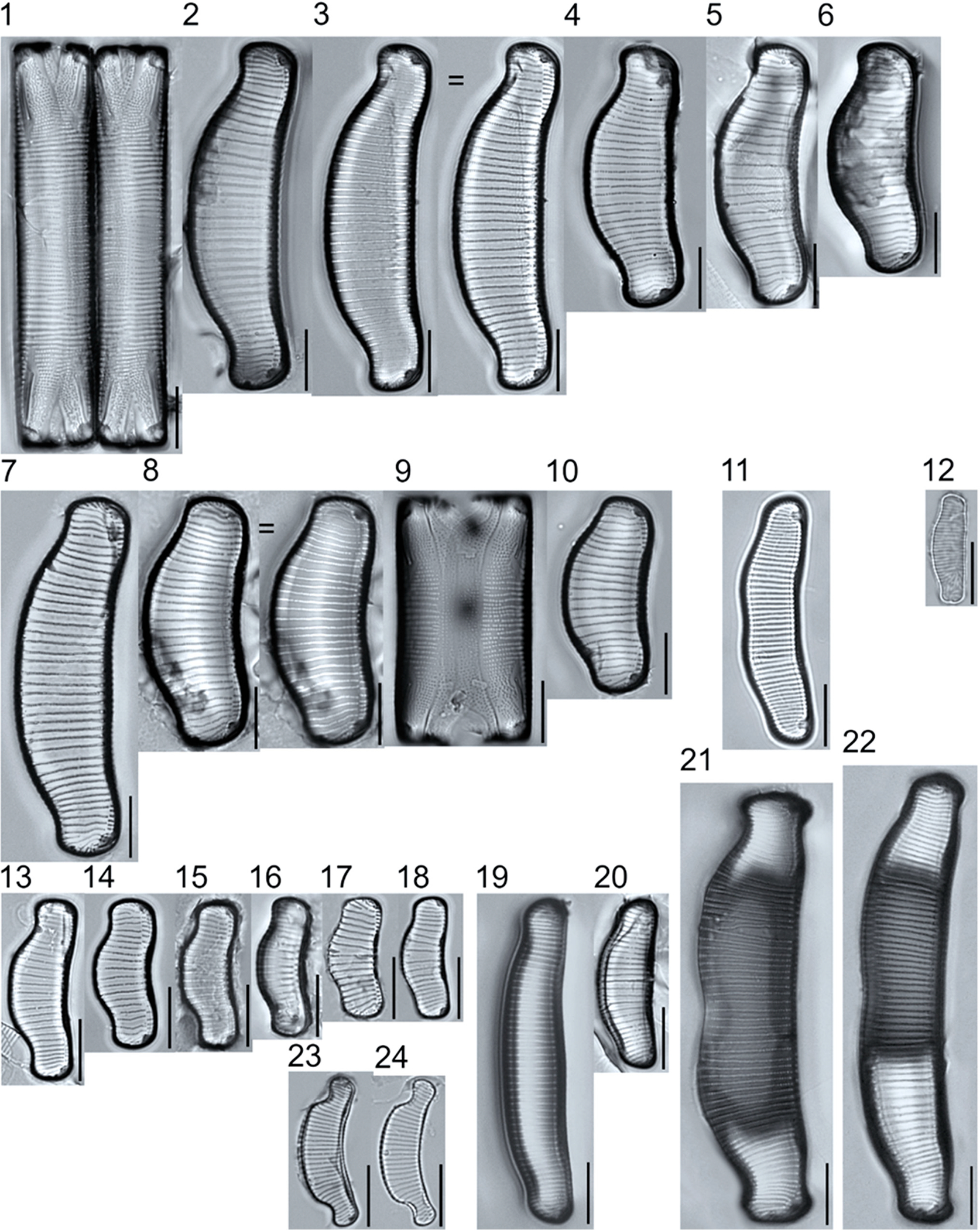
Light micrographs of eunotioid taxa. Figs. 1–6: *Eunotia praerupta*. Figs. 7–10: *E. praerupta* var. *inflata*. Figure 11: *E. dorofeyukae*. Figure 12: *E*. sp.1 APP. Figs. 13–18: *E. curtagrunowii*. Figs. 19–20: *E. arcus*. Figure 21: *E. superbidens*. Figure 22: *E. bidens*. Figure 23–24: *E. septentrionalis*. Scale bars: 10 μm.

**Plate 25.**
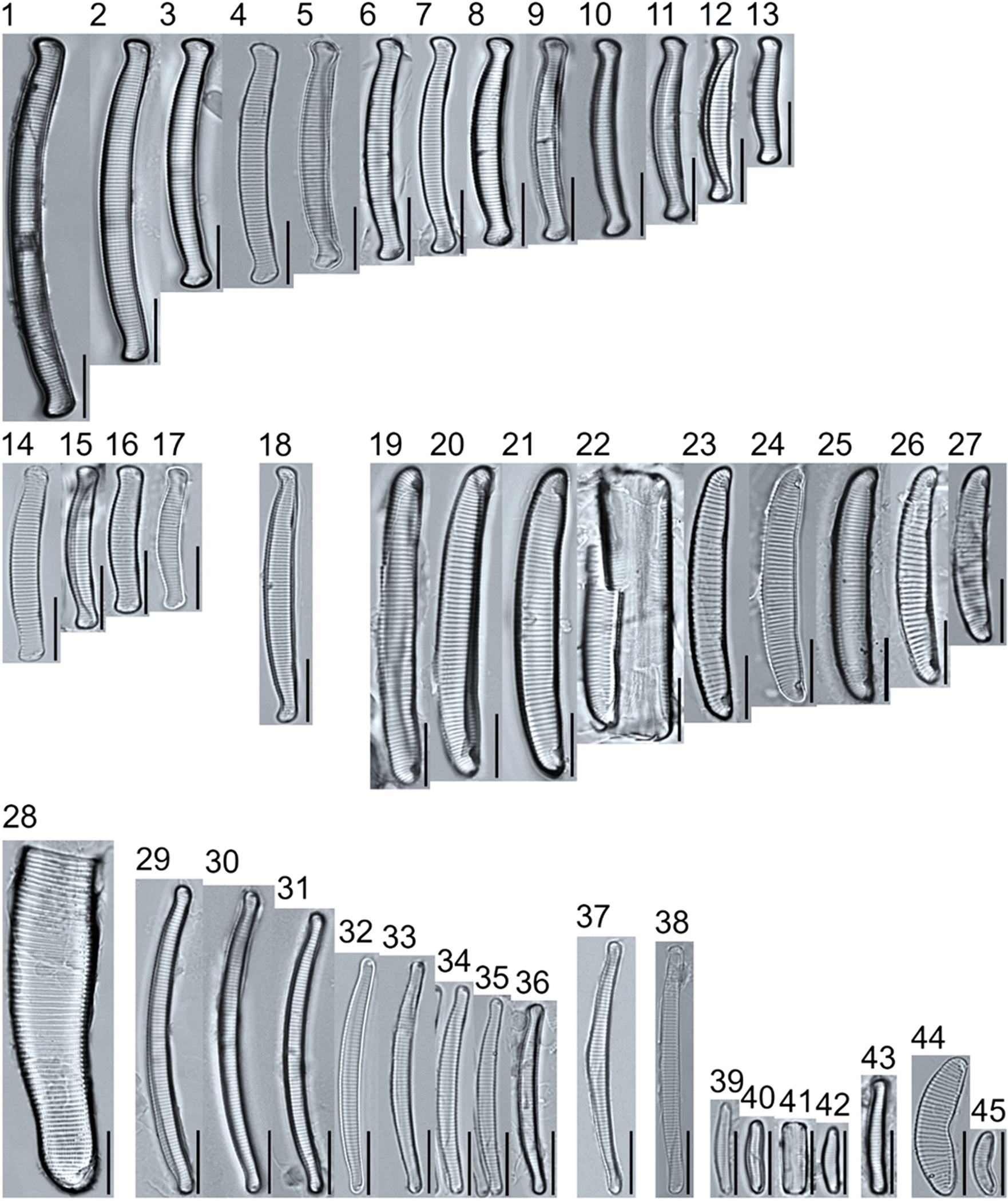
Light micrographs of eunotioid taxa. Figs. 1–13: *Eunotia neocompacta*. Figs. 14–17: *E. neocompacta*. var. *vixcompacta*. Figure 18: *E. superpaludosa*. Figs. 19–27: *E. sedina*. Figure 28: *E*. cf. *maior*. Figs. 29–36: *E. paludosa*. Figure 37: *E*. cf. *trinacria*. Figure 38: *E*. cf. *intermedia*. Figs. 39–42: *E*. cf. *rushforthii*. Figure 43: *E*. sp.3 APP. Figs. 44–45: *E*. sp.4 APP. Scale bars: 10 μm.

The most speciose genus found, *Eunotia*, had the greatest number of distinct morphological forms across all peatlands, with the greatest concentration occurring in the poor fen. For example, Plates 19–25 show 45 OTUs. Of these morphological groupings, 11 were assigned cf. designations and 4 remained at a genus-level designation. The species most frequently encountered in the poor fen survey were *Eunotia naegelii* Migula 1905 (p. 205) and *Eunotia mucophila* (Lange-Bertalot, Nörpel-Schempp, and Alles) Lange-Bertalot 2007 (p. 111).

The Sørensen coefficient was used based on presence–absence data across fen types revealing 41% of species were similar between the rich and moderate fen, 37% were similar between the moderate and poor fen, and only 27% were similar between the rich and poor fens. Comparison of the taxa encountered in this study (Table 2) with other recently published taxa lists revealed 78% dissimilarity with recently recorded diatom species from floras developed for selected southeast rivers in the United States [9], 60% dissimilarity with species from the continental United States checklist [14], and 53% dissimilarity with the species checklist of diatoms from the northwest United States [52]. When compared to the conservation status of taxa in Germany [25], 47 of the 183 fen species encountered in this study (Table 2) matched those considered near threatened (V) or more imperiled status (R, G, 3, 2, or 1).

### 3.2. Red List and Rare Taxa

For selected taxa identified as rare, we provide references used for identification, morphological features within the bounds of the specimens encountered in this study, ecological information, and known distribution records. The taxa detailed below were chosen based on their status in the diatom Red Lists (discussed below) for Germany [24,25] and/or lack of listing in the checklist of diatoms from the continental U.S. [14] and/or the checklist of diatoms from the northwest U.S. [52]. For 44% of the species encountered in our study of these three Alaskan fens, the German Red List had not evaluated their status.

#### *Encyonema neogracile* Krammer 1997 (pp. 177–178).

##### Synonym:

*Encyonema gracile* Rabenhorst 1853 (p. 25, pl. 10, Figure 1). Reported as *Cymbella lunata* Patrick and Reimer 1975 (p. 46, Plate 7, Figs. 11–14); Reported as *Cymbella gracilis* Krammer and Lange-Bertalot 1986 (p. 308, Figure 120: 3–5).

##### Observations: (Plate 5, Figs. 16–29)

The valves are 31.5–44.7 μm long and 4.7–6.3 μm wide, and stria density is 11–15 in 10 μm. Valves are asymmetric about the longitudinal axis, being narrowly cymbelloid, with a moderately arched dorsal margin and weakly convex to flat ventral margin. The apices are narrowly rounded, the raphe is positioned laterally, with proximal raphe ends deflecting dorsally terminating into central pores, and the distal raphe ends curve ventrally. Striae are parallel to slightly radiate, being slightly less dense on the dorsal side and the shorter ventral stria become slightly convergent near the apices.

##### Distribution:

In the United States, Bahls [52] reported 169 prior records (CA, ID, MT, OR, WA, WY) in the Montana Diatom Database and Bahls [53] reports it as widespread (in waters low in nutrients, electrical conductance, and having circumneutral pH) and common in lakes, fens, and mossy seeps in the mountains of the northwest United States. This taxon is reported as presumed endangered [24] and reported as threatened [25] but was not uncommon in our samples from the fens of Alaska.

#### *Encyonema paucistriatum* (Cleve-Euler) D. G. Mann 1990 (p. 667).

Reported as *Cymbella paucistriata* Krammer and Lange-Bertalot 1986 (p. 305, pl. 119, Figures 14–16); Cleve-Euler 1934 (p. 77: pl. 5, Figure 127).

##### Observations: (Plate 5, Figs. 1–14)

The valves are 22.1–42.8 μm long and 5.4–6.5 μm wide, and stria density is 8–11 in 10 μm. The valve outline is lunate with a flat to slightly tumid ventral margin, moderately arched dorsal margin, and rounded apices. Striae are slightly radiate to parallel, and density is variable with some specimens having irregularly spaced striae (Plate 5, Figure 12).

##### Distribution:

In the US, this taxon was not listed in recent checklists [14,52]. It was described from Finnish Lapland [54] and has been reported from northern Sweden and the European Alps in oligotrophic waters [38] and wetland habitats (pH: circumneutral; conductivity: low; nutrients: low) on the tundra in Nunavut, Canada [53]. This taxon is reported as highly threatened and rare [25]; however, it was not uncommon in our samples from the fens of Alaska.

#### *Encyonema procerum* Krammer 1997 (p. 169, pl. 32: Figures 9–19)

Reported as *Encyonema droseraphilum* Bahls et al. 2013 (p. 36, Figures 3–10).

##### Observation: (Plate 5, Figure 34)

The valve is 31.4 μm long and 6.8 μm wide, with a stria density of 8–11 in 10 μm dorsally and 12–13 in 10 μm ventrally. The valve is cymbelloid, with a weakly convex to flat ventral margin and a moderately arched dorsal margin. Striae of the dorsal side are slightly radiate to parallel, being slightly less dense than ventral striae, which are short and parallel to convergent nearing the apices. The proximal raphe ends are inflated slightly and deflected dorsally, and distal raphe ends are curved towards the ventral margin.

##### Distribution:

In the United States, Bahls et al. [55] reported it (as *E. droseraphilum*) from a floating mat fen (pH: 6.7; conductance: 257 μS/cm) and a shallow lake in the forested mountains of northwestern Montana. It was originally described from the freshwaters of Heinersreuth in Upper Franconia in Bavaria, Germany [56]. This taxon was not reported in Lange-Bertalot [24], reported as extremely rare, threatened with extinction in the German Red List [25], and in our initial screening for this voucher production, it was only encountered once.

#### *Eunotia naegelii* Migula 1905 (p. 203).

Available in Lange-Bertalot et al. 2011 (p. 167: pl. 21, Figures 1–23; pl. 22, Figures 1–13); Furey [57].

##### Observations: (Plate 22, Figs. 1–5)

The valves are 103.8–123.1 μm long and 2.7–3.1 μm wide, with a stria density of 15–17 in 10 μm near the center and 17–20 in 10 μm in the apices. Valves are moderately arched with dorsal and ventral margins nearly parallel in the center and narrowing to slightly dorsally deflected, barely inflated apices. The distal raphe fissures curve onto the valve face, bending 180° and continuing a short distance toward the proximal raphe ends.

##### Distribution:

In the United States it was reported in the Laurentian Great Lakes [58], in the Northwest checklist from California, Oregon, and Montana [52], and detected in the South Saluda River, Cleveland, South Carolina [9]. It was reported in a checklist for the British Isles and adjoining coastal waters [59], a checklist of the Gulf of Mexico and coastal waters [60], and as infrequent in the Holarctic, Eurasia, and North America being abundant in few places [46] (see discussion for autecology). This taxon is reported as at risk [24], reported as rare and threatened [25], but was not uncommon in our samples from the fens of Alaska.

#### *Gomphonema lagerheimii* A. Cleve 1895 (p. 22, pl. 1: Figure 15).

Specimens with similar morphology were reported as *Gomphonema hebridense* Gregory 1854 in Cantonati et al. 2017, Bahls [52], and Bahls et al. [55], but none of the specimens bear a resemblance to Gregory’s (1854) original drawings of *G. hebridense*. The specimens do match the original description and drawing of *G. largerheimii* A. Cleve 1895.

##### Observations: (Plate 6, Figs. 28–46)

The valves are 33–55 μm long and 4.3–7 μm wide, and stria density is 12–18 in 10 μm. The valve outline is nearly symmetrical about the longitudinal axis with a slightly tumid center and a linear-lanceolate shape, having one stigma lying at the end of a short median stria in the central area, appearing slightly cymbelloid in partial valve view.

##### Distribution:

In the United States, Bahls [52] (as *G. hebridense*) reported low numbers in nine streams (pH: 6.8; mean conductance: 247 μS/cm) in western Montana and western Oregon and Bahls et al. [55] (as *G. hebridense*) detected populations in the floating mat fens of the Indian Meadows Research Natural Area, 90 km northwest of Helena, Montana. In Austria, Germany, and Finland it has been reported as a northern-alpine species [61,62]. This taxon is reported as declining [24], reported as near threatened [25], but was not uncommon in our samples from the fens of Alaska.

#### *Kobayasiella parasubtilissima* (Kobayasi and Nagumo) Lange-Bertalot 1999 (p. 268).

##### Synonym:

*Navicula parasubtilissima* Kobayasi and Nagumo 1988 (pp. 245, 247, Figures 19–37).

##### Observations: (Plate 9, Figs. 1–10)

The valves are 29.8–34.6 μm long and 4.1–4.8 μm wide. Stria density was not resolvable in LM but has been reported as 40–42 in 10 μm [55]. The valve outline is linear-lanceolate with slightly convex margins, apices are capitate, and the axial area is narrow.

##### Distribution:

In the United States, it has been reported in low alkalinity lakes in the Northeast [63], 19 lakes and streams (mean pH: 7.5; mean conductance: 116 μS/cm) in Montana and Washington [52] (as *Kobayasiella subtilissima*), and detected populations in floating mat fens near Helena, Montana [55]. This taxon has also been reported from Lake Imandra, Russian Lapland, Cleve [64] (p. 37); high moors in the Alps and Scandinavia, in association with *Sphagnum* species [38]; and lakes in northern Québec and Labrador [65] (as *Navicula parasubtilissima*). This taxon is reported as declining [24], rare, and near threatened [25], but was not uncommon in our samples of the fens of Alaska.

#### *Stauroneis heinii* Lange-Bertalot and Krammer 1999 (p. 91, pl. 27, Figures 1–4).

##### Observations: (Plate 16, Figure 1) T

The valve is 157.6 μm long and 30.2 μm wide, with a striae density of 15–16 in 10 μm and areolae number 16–17 in 10 μm. The valve outline is elliptic lanceolate with protracted ends and external proximal raphe fissures are strongly inflated and strongly curved.

##### Distribution:

In the United States, it has been reported from Alaska [66] and western Montana, where it prefers slightly acidic to circumneutral waters with low concentrations of electrolytes [52] and in the floating mat fens of the Indian Meadows Research Natural Area, 90 km northwest of Helena, Montana [16,55]. It has been reported as bipolar, being detected from Siberia [67], Greenland [68], the Andes Mountains from Venezuela to Patagonia [69], South Georgia Island [70], and the Canadian Arctic [71]. It was not encountered in the Kociolek [14] contiguous United States checklist; therefore, it was first reported for the contiguous United States in Bahls [16,54]. This taxon was not reported in the German diatom Red Lists [24,25] and in our initial screening for this voucher production, it was only encountered once.

#### Stauroneis indianopsis Bahls 2010 (pp. 85–86).

##### Observations: (Plate 16, Figure 3)

The valve is 124 μm long and 24.8 μm wide, with a stria density of 16–17 in 10 μm, and 16–18 areolae in 10 μm. The valve is linear-lanceolate, the apices are slightly protracted, the axial area narrow, the striae radiate, the stauros narrow (linear or slightly expanded toward the valve margins), the raphe fissures lateral, the proximal ends strongly curved and weakly inflated, and the terminal raphe fissures are hooked.

##### Distribution:

In the United States, Bahls [16] described it from floating mat fens from the Indian Meadows Research Natural Area, 90 km northwest of Helena, Montana [55], and from a small lake (pH: 7.5; conductance: 10 μS/cm) in Missoula County, Montana [16]. It was not encountered in the Kociolek [14] contiguous United States checklist; therefore, it was first reported for the contiguous United States in Bahls [16], and this may be the first report for Alaskan fens. This taxon was not reported in the German diatom Red Lists [24,25] and in our initial screening for this voucher production, it was only encountered once.

#### *Stauroneis subborealis* Bahls 2010 (pp. 151–152).

##### Observations: (Plate 16, Figure 5)

The valve is 106.6 μm long and 16.8 μm wide, with a stria density of 18–19 in 10 μm and 19–21 areolae in 10 μm. The valves are linear-lanceolate, the apices are protracted and broadly rounded, the axial area is narrow (slightly widening near the central area), the striae radiate, the stauros narrow (slightly expanded toward the valve margins), the raphe fissures lateral, the proximal ends curved and inflated, and the terminal raphe fissures are hooked.

##### Distribution:

In the United States, Bahls [16] described it from material collected at Indian Meadows Research Natural Area and encountered it in a few ponds, fens, and small lakes (appearing tolerant of a wide range of pH and low to moderate concentrations of electrolytes) in western Montana [55]. It was not encountered in the Kociolek [14] contiguous United States checklist; therefore, it was first reported for the contiguous United States in Bahls [16] and this may be the first report for the Alaskan fens. This taxon was not reported in the German Red Lists [24,25] and in our initial screening for this voucher production, it was only encountered once.

#### *Stenopterobia delicatissima* (F. W. Lewis) Brébisson ex van Heurck, 1896 (p. 374; pl. 1, Figures 19–51).

##### Synonym:

*Surirella delicatissima f. delicatissima* Lewis 1864. Available in Krammer and Lange-Bertalot 1988 (2/2, p. 210, pl. 170: 5, 6; pl. 173: 1–8; pl. 174: 1–12).

##### Observations: (Plate 7, Figs. 3–12)

The valves are 53.2–76.0 μm long and 4.0–4.6 μm wide, stria density is 18–28 in 10 μm, and fibulae is 4–7 in 10 μm. Valves are lightly silicified and linear-lanceolate, parallel to slightly convex towards the center, then tapering into attenuate apices. Striae are parallel throughout, slightly off-set from one another at the central sternum which may be difficult to discern in light microscopy (LM). The raphe is circumferential, raised onto a clearly discernable keel.

##### Distribution:

In the United States, it has been reported in Kociolek [14] referencing its detection in southern Alabama swamps (pH: ~5.0) colonizing the mucilage of *Ophrydium* where it was found to be abundant [72]. Siver et al. [73] examined materials from the type locality, Saco Pond (an acidic spring-fed waterbody), New Hampshire, and it has been reported from Montana [52]. It has been reported as widespread and cosmopolitan in humic acidic waters but rare in grassy plains [39]. This taxon is reported as threatened [24], rare, and highly threatened [25], but was not uncommon in our samples from the fens of Alaska.

## 4. Discussion

### 4.1. Assemblage Analysis

As anticipated, we found diatom assemblages consistent with recent studies [22,49,55] in other high-latitude wetlands containing characteristic minerotrophic, acidophilous, and epiphytic taxa such as *Eunotia*, *Gomphonema*, and *Pinnularia*. Bahls [55] found intact relict assemblages comprised of 49 taxa that included arctic, sub-arctic, and boreal diatom species in two undisturbed floating-mat fens in Montana. Of those, 27 are considered at risk or declining according to the diatom Red List developed in Germany [25] which they inferred to be appropriate designations for the cold-loving, rare, northern fen diatoms within the United States. Here, we found similar results, with 46 of our 184 species matching those listed as near threatened, extremely rare, threatened, at risk or declining, highly threatened, or threatened with extinction in the Red List developed in Germany [25]. Many diatom floristic studies of peatlands in North America have frequently documented rare or new species [74,75], yet information on the biodiversity of peatland diatoms remains sparse compared with other aquatic environments [76]. The northern boreal region has been shown to possess a unique diatom flora, with characteristic taxa and high species richness in the rivers, lakes, and streams across Alaska [48,49]. The present study also updates and expands regional knowledge building on Foged’s 1981 treatment of Alaskan diatom flora to include a gradient of peatlands that are home to many rare, threatened, and potentially new species of diatoms [50].

*Tabellaria flocculosa*, which was common in all peatlands in our study, is cosmopolitan, often found in a wide range of water types (ranging from acidic to alkaline), frequently occurring in northern latitudes, and is commonly found in lakes, running water, and peat bogs [4]. Over time, authors distinguished *T. flocculosa* in several ways owing to its high variability in morphological forms [77]. For example, Knudson [78] described four varieties based primarily on colony morphology, and Koppen [79] described three “strains” based on size range and autecology. Our understanding of the species concept follows the variability noted in the United States, which includes strains III, IIIp, and IV together when defining *T. flocculosa* [78,79]. Reported as abundant only in low-nutrient, soft waters [47], *T. flocculosa* is considered “not threatened” and is moderately common [25].

In the rich fen, populations of *Eunotia pseudoflexuosa* and *Tabellaria fenestrata* frequently occurred in addition to the high density of *T. flocculosa*. Foged [50] reported *E. pseudoflexuosa* as halophobic and acidophilic in three samples from Alaska. Additional distribution records detected *E. pseudoflexuosa* in Central Africa, South Africa, Europe, Canada, and a *Sphagnum* bog complex in Russia [46]. As a known associate of *T. flocculosa*, *T. fenestrata* often occurs in lower relative abundance [80]. *T. fenestrata’s* described ecological range varies in the literature; however, detection in circumneutral waters, especially mesotrophic-eutrophic ponds and lakes, occurs often [81]. *T. fenestrata* can be planktonic [40] but is often found growing attached to hard substrates and vegetation such as *Sphagnum* [78–80]. *T. fenestrata* is distinguished by colonies that form long straight chains, two to four septa in girdle view, and approximately equal width inflations [78–80], and is rarely observed in stellate formations or zig-zag colonies.

The most frequently encountered species in the moderate fen, *Pinnularia pulchra* and *Eunotia pseudoparallela*, are described as epipelic in oligotrophic waters with low electrolyte content in East Greenland and northern Finland and are reported as absent from Europe [44]. Han et al. [82] found *P. pulchra* as one dominant diatom species in herbaceous peatlands in the northern Greater Khingan Mountains, China, tolerant of neutral-alkaline habitats. Likewise, the moderate fen conditions align as suitable to support *P. pulchra*. Similarly, *E. pseudoparallela* rarely occurs in the Holarctic, central Europe, or southern Europe, yet appears abundant in Scandinavian minerotrophic peatlands or comparable moderately acidic, electrolyte-poor habitats [46].

As expected, species of *Eunotia*, including *E. naegelii* and *E. mucophila*, were common in the acidic waters of the poor fen. Similar to the majority of *Eunotioid* taxa, the autecological preferences of *E. naegelii* are dystrophic, nutrient-poor, moderately acidic fens, lakes, and springs with low specific conductivity [46]. *E. mucophila,* reported as highly abundant in *Sphagnum* peat bogs and dystrophic lakes, remains infrequently reported in the Holarctic flora, Eurasia, and North America [46]. In the United States, *E. mucophila* has been observed in the Adirondack Mountains of New York [63]; South Carolina [83]; Cape Cod, Massachusetts [84]; and in the acidic lakes of Acadia National Park, Maine [85]. The German Red List reported *E. mucophila* as rare and under ‘Threat of Unknown Extent’ because the available information is not sufficient to allow a precise assignment to categories one to three [25].

We encountered a few diatom valves we consider to be teratological forms (i.e., abnormal physiological development) (Pl. 5, Figure 15. *E*. sp.1 APP; Pl. 5, Figure 46. *E*. sp.2 APP; Pl. 24, Figure 12. *E*. sp.1 APP). Deformities are observed in natural diatom assemblages, but their prevalence is relatively low (<0.5%) [86]. Taxa disposed to teratological forms (e.g., *Fragilaria, Eunotia*) under natural conditions may falsely indicate contamination; thus, abnormalities in these genera alone within an assemblage should be interpreted accordingly [87]. The minimal anthropogenic impacts on the studied peatland complex suggest these teratological forms do not indicate contamination. Just as we included the few valves of rare taxa, we chose to include the few teratological forms (rather than exclude them, which is typical) to reflect the full diatom assemblage composition.

### 4.2. Ecology Inferred

Historically, diatoms were underexplored in ecological monitoring studies of peatlands despite being a commonly employed tool in other environments [88]. Diatoms occur in abundance in surveyed peatlands, including those reported from the early work of Reimer [74] and Stoermer [75]. Later, Kingston [89] subsequently identified characteristic peatland diatom assemblages concluding diatoms are sensitive to microhabitat conditions (e.g., water table position, macro-vegetation type, and trophic status) and good indicators of environmental gradients in peatlands. More recently, diatoms have been identified as one of the most widely represented algal groups in peat bogs and fens [29,90] and were found to alter their assemblage composition significantly, in kind with subtle shifts in moss species assemblage composition [91]. Diatoms readily respond to changes in pH and moisture content [92]. We report differences in diatom assemblage composition among our peatlands to further emphasize their potential biomonitoring power applied within these wetland environments. Furthermore, our findings of approximately 10% of the documented species across all fens being potentially new to science highlight the uniqueness of peatland diatom communities.

The distinctiveness of peatland habitats (i.e., rarity, stability, and extreme conditions) explain the unique vascular plant flora, as well as the high concentrations of rare species restricted there [34]. Similarly, these unique conditions support new species and rare diatoms found in peatland floristic studies [22,55,74,75]. Some diatom species capitalize on the changing environment in boreal wetlands [93] and are sensitive enough to use as a proxy to assess the magnitude of past hydrological changes [94]. Diatom species that are often rare and strictly bound to fens have adapted to withstand selection pressures such as extended periods of desiccation, thermal fluctuations, low nutrient concentrations (particularly nitrogen), and low pH [55]. For example, genera such as *Eunotia* and *Pinnularia*, commonly observed in this study, exhibit higher species diversity in wetland environments [95]. The minimal number of species from the order Centrales was expected owing to the shallow fen waters which prevent suspension of planktonic taxa [4]. The unique conditions typical of peat bogs and fens [22,55] likely supported a number of the rare taxa observed in this study. This diatom voucher flora, produced as a practice in taxonomic transparency, will support the use of diatoms as bioindicators in these distinctive wetlands.

## 5. Conclusions

Renewing commitment to the development of region-specific voucher floras is imperative to better understand the biodiversity of diatoms, the ecosystem services they provide (e.g., oxygen production, foundation of the food web), and their application in solving ecological problems [14]. The way in which diatoms will be employed as bioindicators in Alaska peatlands will depend on several factors, such as the questions being asked, along with the need to balance precision, speed, and fiscal responsibility. For example, studies interested in exploring biodiversity may consider different counting methods to capture more taxa [96], and ongoing work will use this diatom flora to build a predictive model. Rare diatom taxa can be perceived as noise during data analysis for ecological bioassessments. This perception can lead to the exclusion of as many as 70% of diatoms in a data set (1028 out of 1461 taxa) prepped for analysis [97]. Therefore, voucher floras that include rare species should be strongly considered during ecological assessment. The data presented here could be used to expand species concepts, distribution records, and autecological information relating certain taxa of diatoms with environmental parameters of fens. Despite diatoms being acknowledged as important for biodiversity/species richness assessments, there is still a need for further investigation into their role as bioindicators e.g., [98] in these unique wetland ecosystems. This voucher flora of the boreal peatlands of interior Alaska is part of a collective effort to provide accessible taxonomic identification resources for localized areas to support wetland conservation.

## Supplementary Material

Supplement1

## Figures and Tables

**Plate 1.**
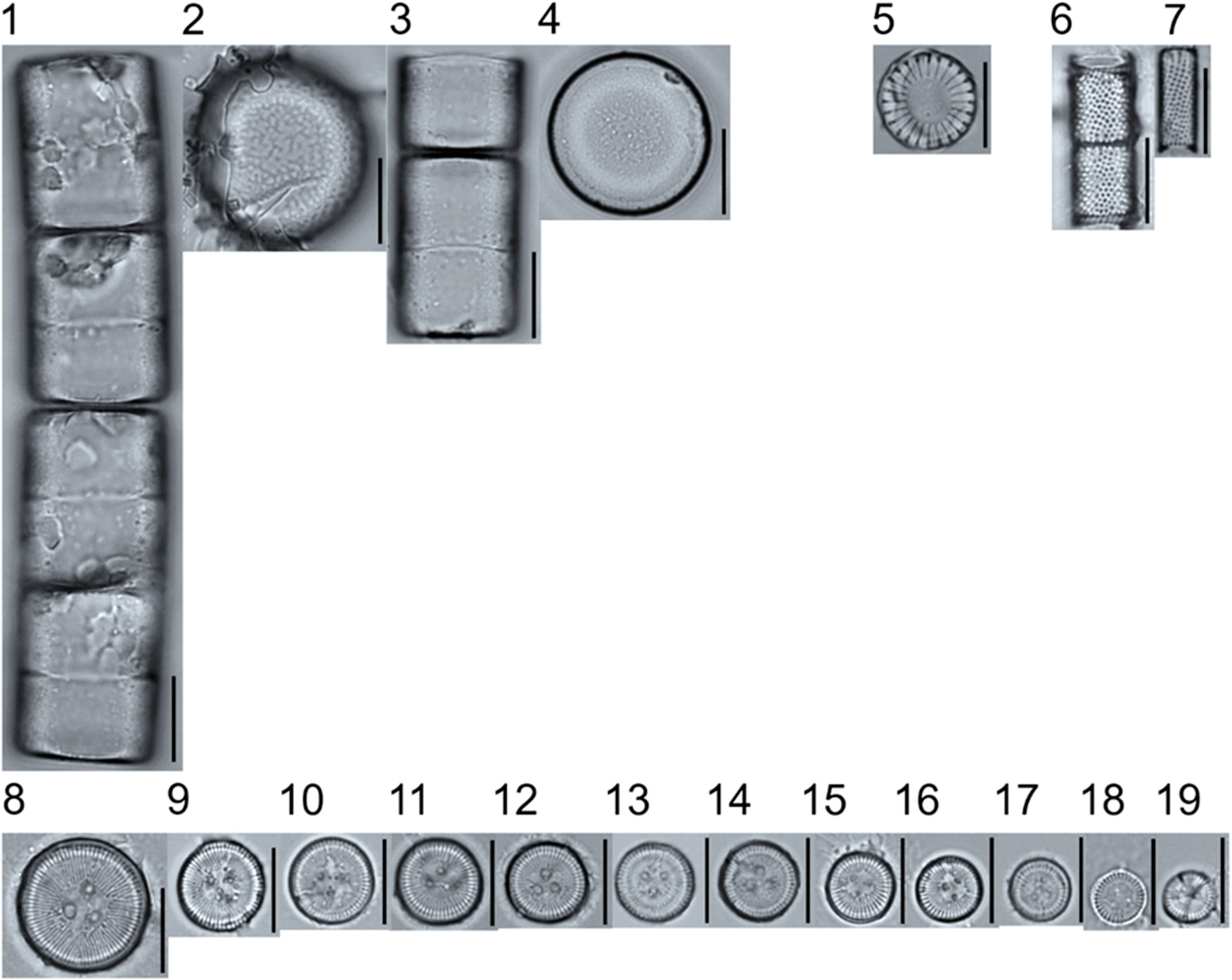
Light micrographs of centric taxa. Figs. 1–4. *Melosira varians*, valve and girdle views. Figure 5. *Stephanocyclus meneghinianus*, valve view. Figs. 6–7. *Aulacoseira ambigua*, mantle views. Figs. 8–19. *Lindavia ocellata*, valve views. Scale bars: 10 μm.

**Plate 3.**
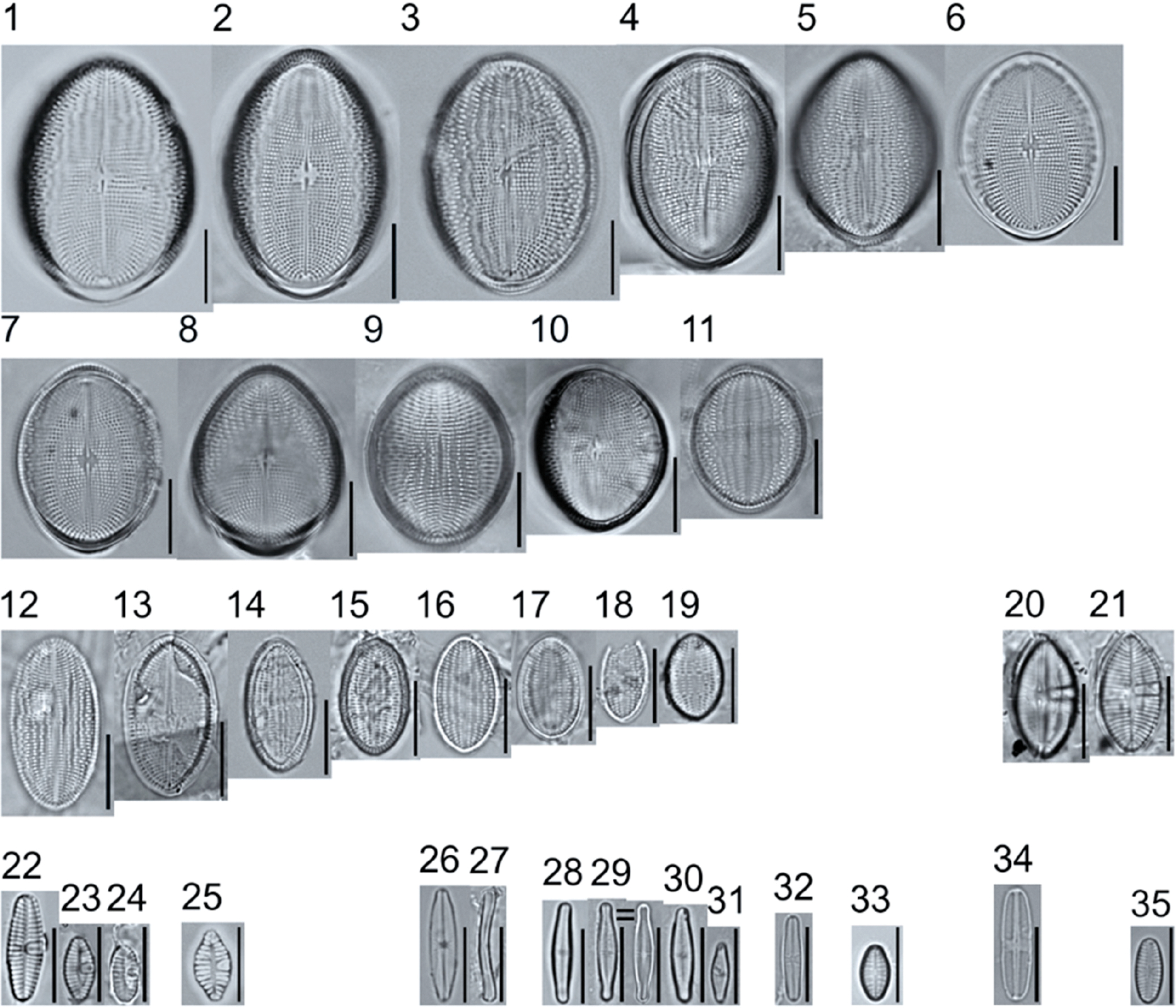
Light micrographs of monoraphid taxa. Figs. 1–11: *Cocconeis pediculus*. Figs. 11–19: *C. placentula sensu lato*. Figs. 20–21: *Skabitschewskia oestrupii*. Figure 22–24: *Planothidium frequentissimum*. Figure 25: *P. rostratoholarcticum*. Figs. 26–27: *Achnanthidium alpstre*. Figs. 28–31: *A*. cf. *gracillimum*. Fig. 32: *A. minutissimum* var. *jackii*. Figure 33: *A*. sp.1 APP. Figure 34: *Rossithidium petersenii*. Fig. 35: *Psammothidium* cf. *microscopicum*. Scale bars: 10 μm.

**Plate 4.**
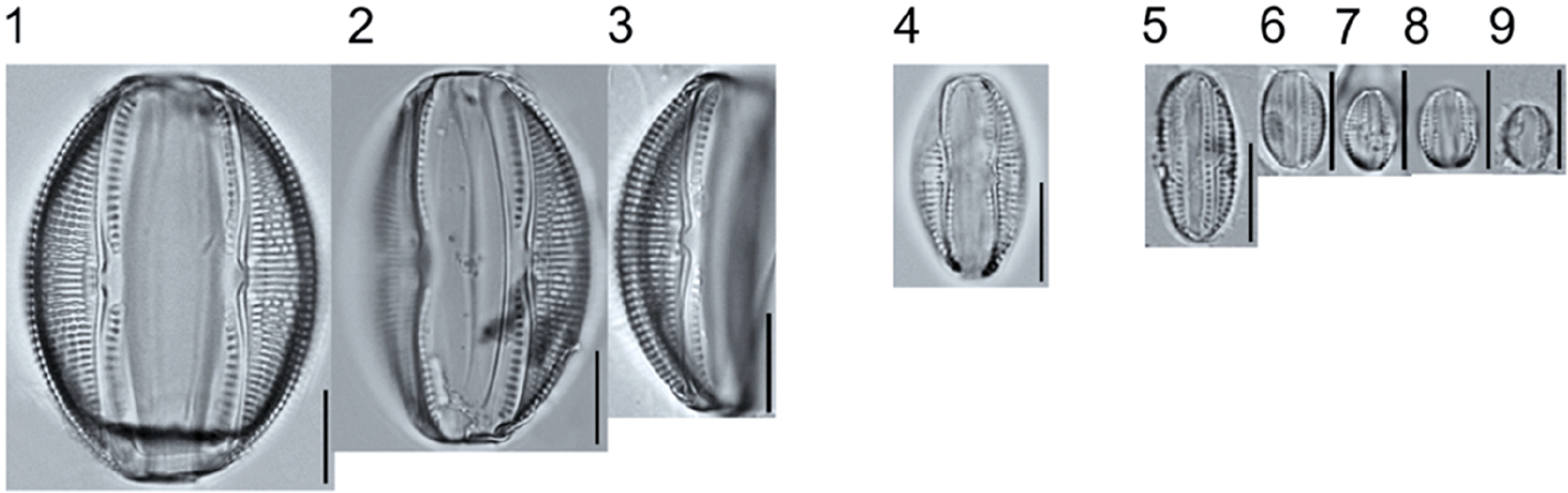
Light micrographs of asymmetric biraphid taxa. Figs. 1–3: *Amphora ovalis*. Figure 4: *A. copulata*. Figs. 5–9: *A. pediculus*. Scale bars: 10 μm.

**Plate 6.**
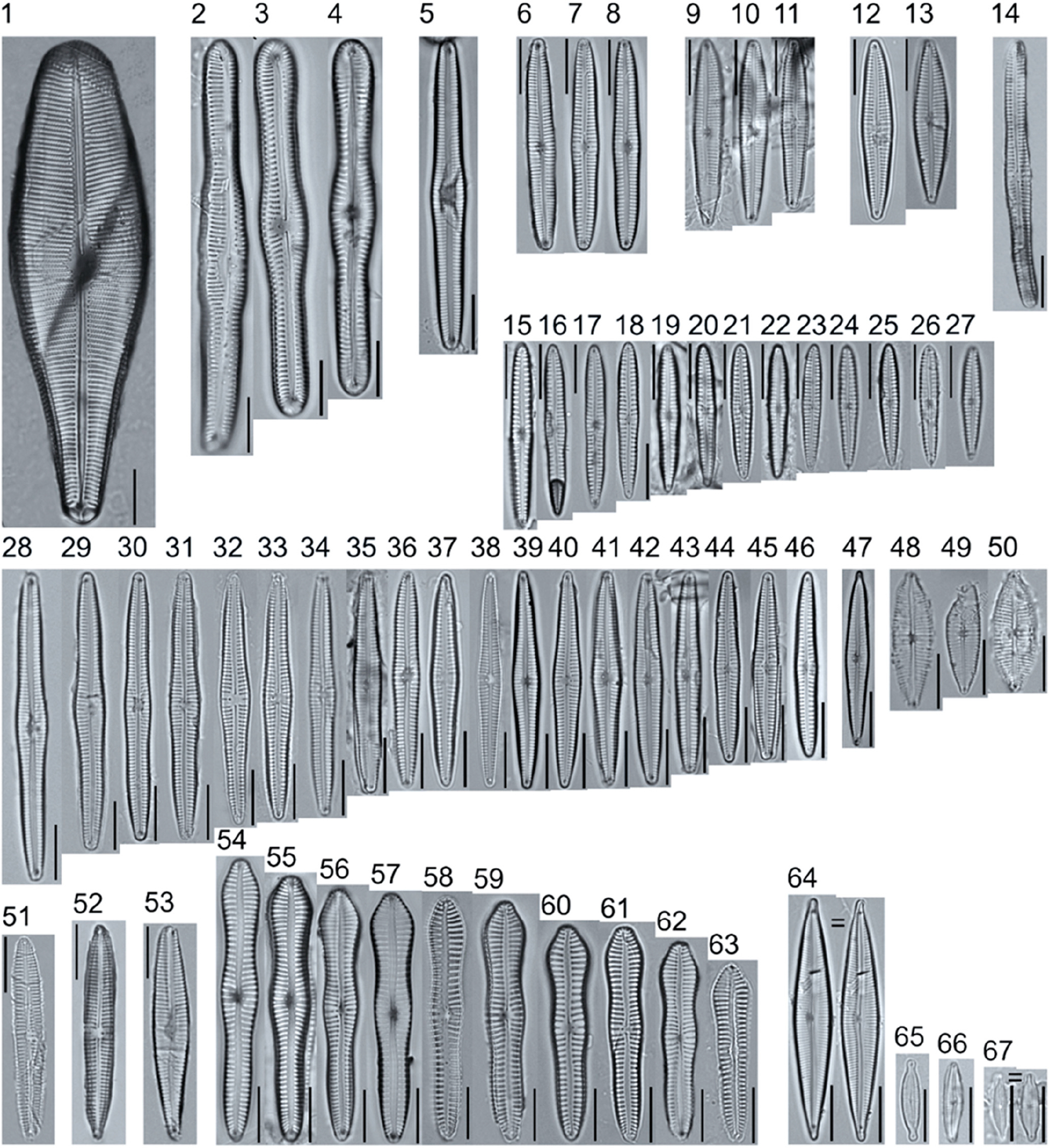
Light micrographs of asymmetric biraphid taxa. Figure 1: *Gomphoneis herculeana*. Figure 2–4: *Gomphonema* sp.1 APP. Figure 5: *G*. sp.2 APP. Figs. 6–8: *G*. sp.3 APP. Figs. 9–11: *G*. sp.4 APP. Figure 12–13: *G*. cf. *parvulum*. Figure 14: *G. laterpunctatum*. Figs. 15–27: *G*. cf. *raraense*. Figs. 28–46: *G. lagerheimii*. Figure 47: *G*. sp.5 APP. Figure 48–50: *G. parvulum*. Figure 51: *G*. sp.6 APP. Figure 52: *G*. sp.7 APP. Figure 53: *G*. sp.8 APP. Figs. 54–63: *G. brebissonii*. Figure 64: *Encyonopsis montana*. Figure 65: *E*. cf. *microcephala*. Figure 66: *E. thumensis*. Figure 67: *E*. cf. *minuta*. Scale bars: 10 μm.

**Plate 7.**
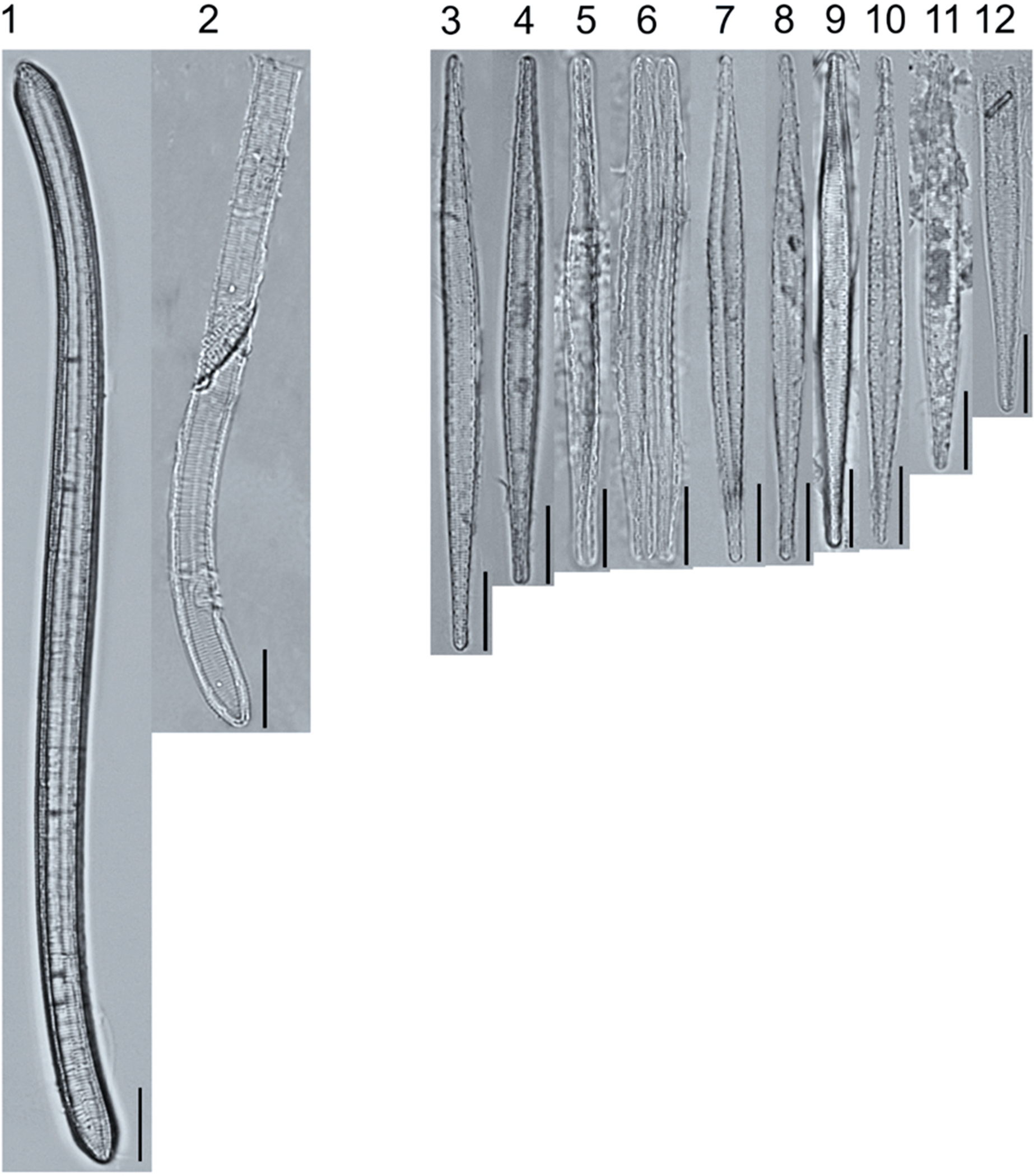
Light micrographs of surirelloid taxa. Figs. 1–2: *Stenopterobia anceps*. Figs. 3–12: *S. delicatissima*. Scale bars: 10 μm.

**Plate 8.**
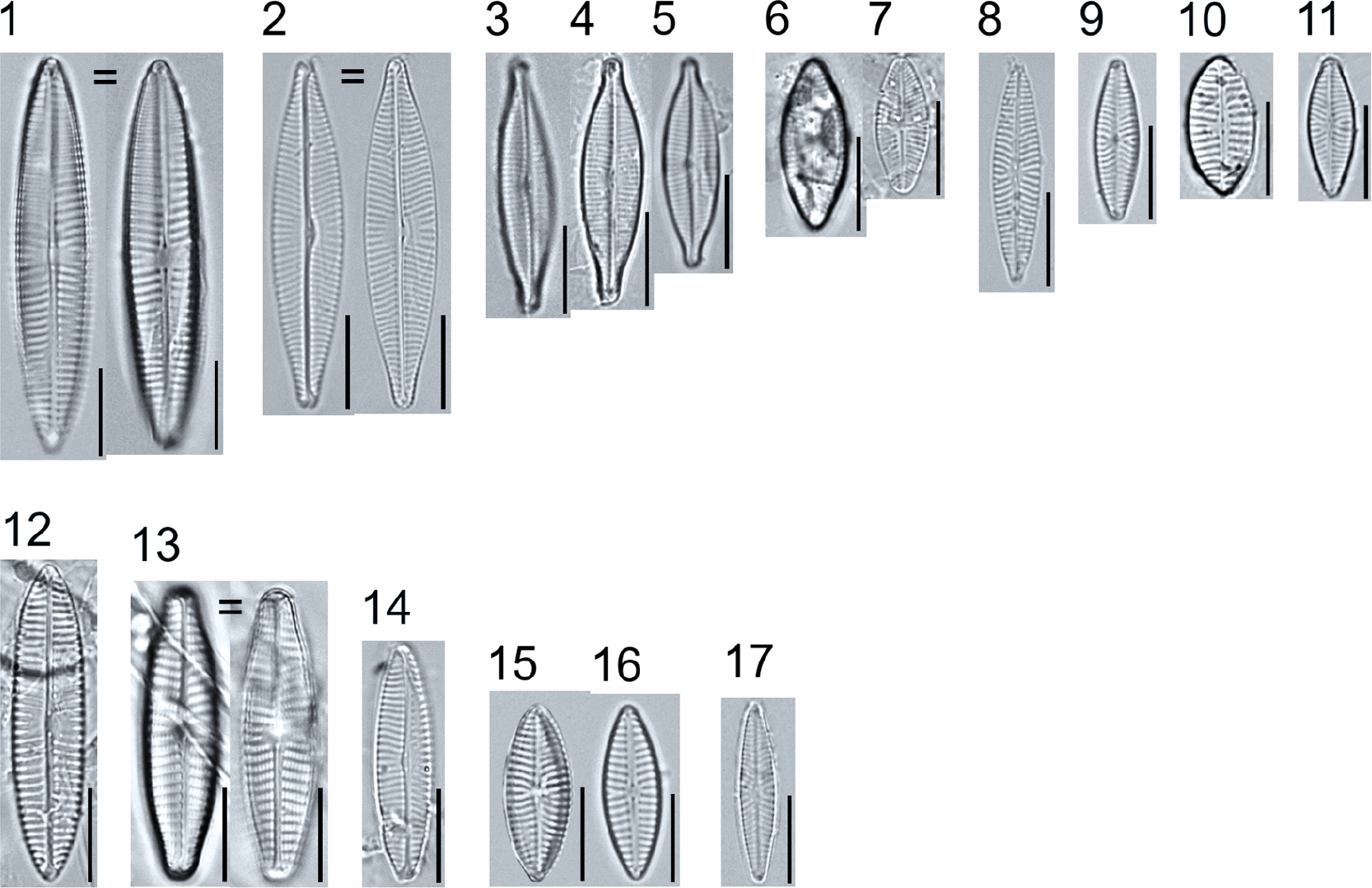
Light micrographs of symmetric biraphid taxa. Figure 1, 12: *Navicula tripunctata*. Figure 2: *N. germanii*. Figs. 3–5: *N. gregaria*. Figs. 6–7: *Diadesmis* sp.1 APP. Figure 8: *Navicula cryptotenella*. Figure 9: *N. metareichardtiana*. Figure 10: *N*. cf. *catalanogermanica*. Figure 11: *N. caterva*. Figure 13: *N*. cf. *streckerae*. Figure 14: *N. erifuga*. Figure 15–16: *N. antonii*. Figure 17: *N. tenelloides*. Scale bars: 10 μm.

**Plate 9.**
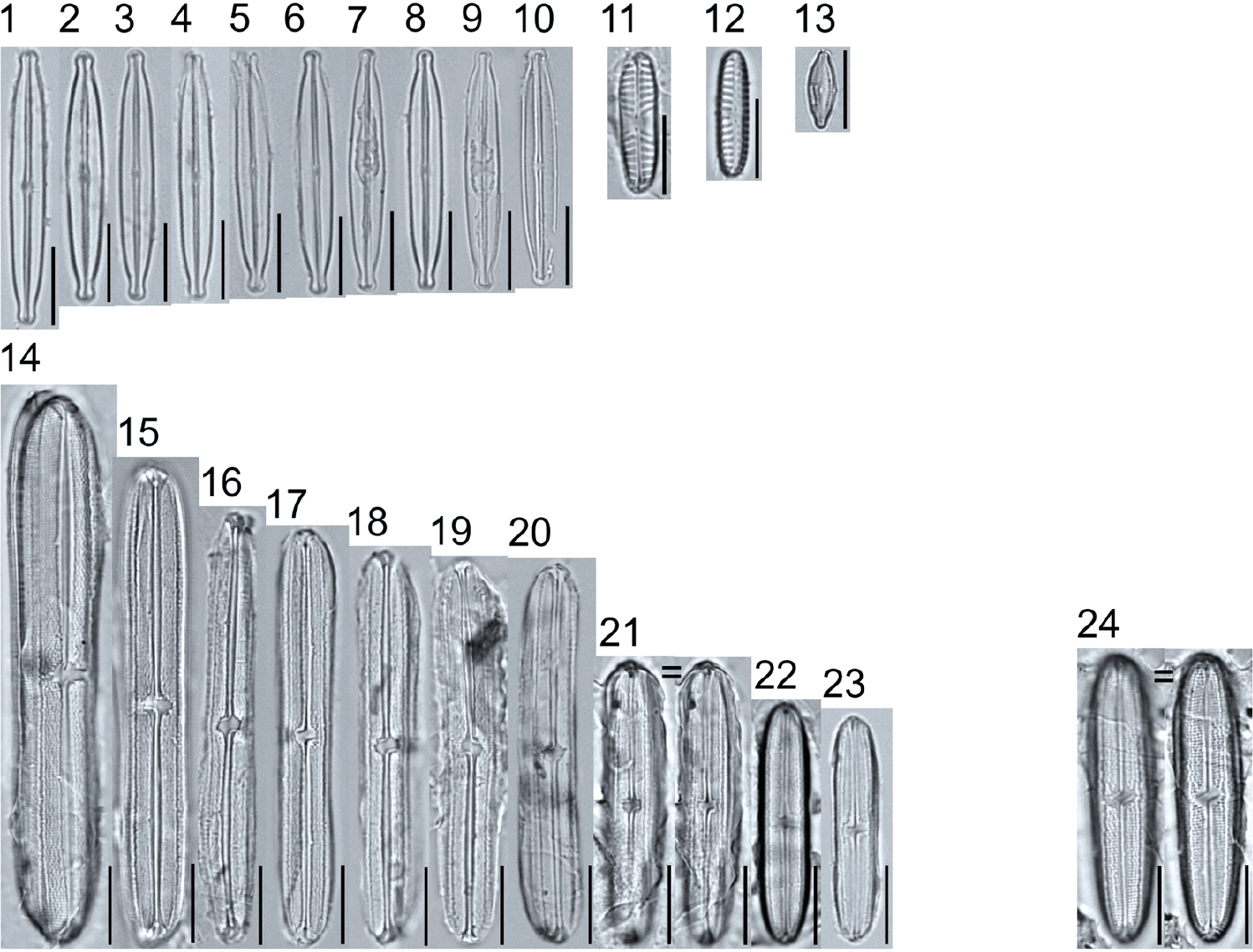
Light micrographs of symmetric biraphid taxa. Figs. 1–10: *Kobayasiella parasubtilissima*. Figure 11: *Hippodonta pseudopinnularia*. Figure 12: *Navicula* cf. *cincta* (see Plate 8 for *Navicula*). Figure 13: *Microcostatus* sp. 1 APP. Figs. 14–23: *Neidium bisulcatum*. Figure 24: *N. bisulcatum* var. *subampliatum*. Scale bars: 10 μm.

**Plate 10.**
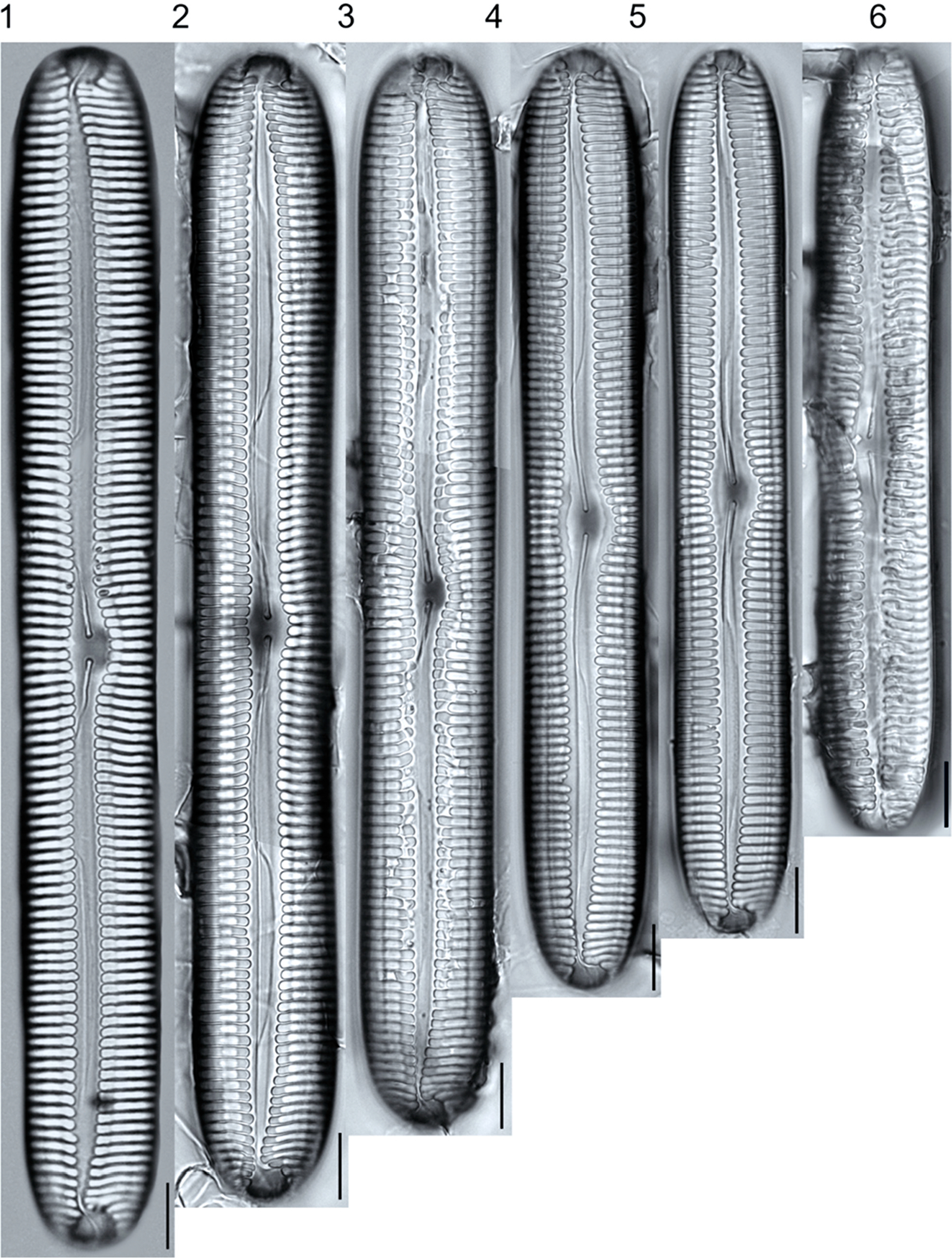
Light micrographs of symmetric biraphid taxa. Figs. 1–6: *Pinnularia neomajor*. Scale bars: 10 μm.

**Plate 13.**
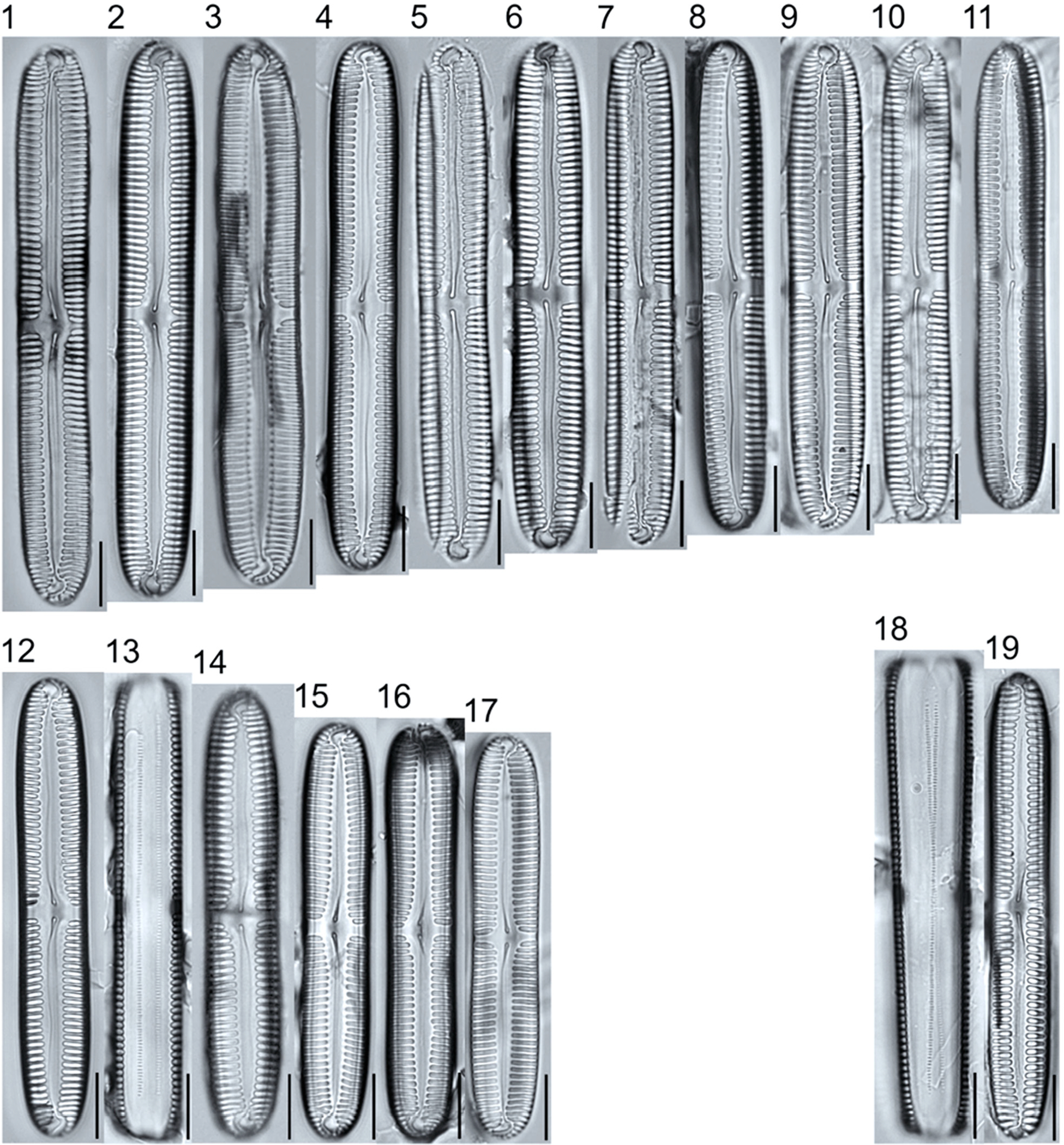
Light micrographs of symmetric biraphid taxa. Figs. 1–17: *Pinnularia aestaurii* var. *interrupta*. Figs. 18–19: *P. aequilateralis*. Scale bars: 10 μm.

**Plate 14.**
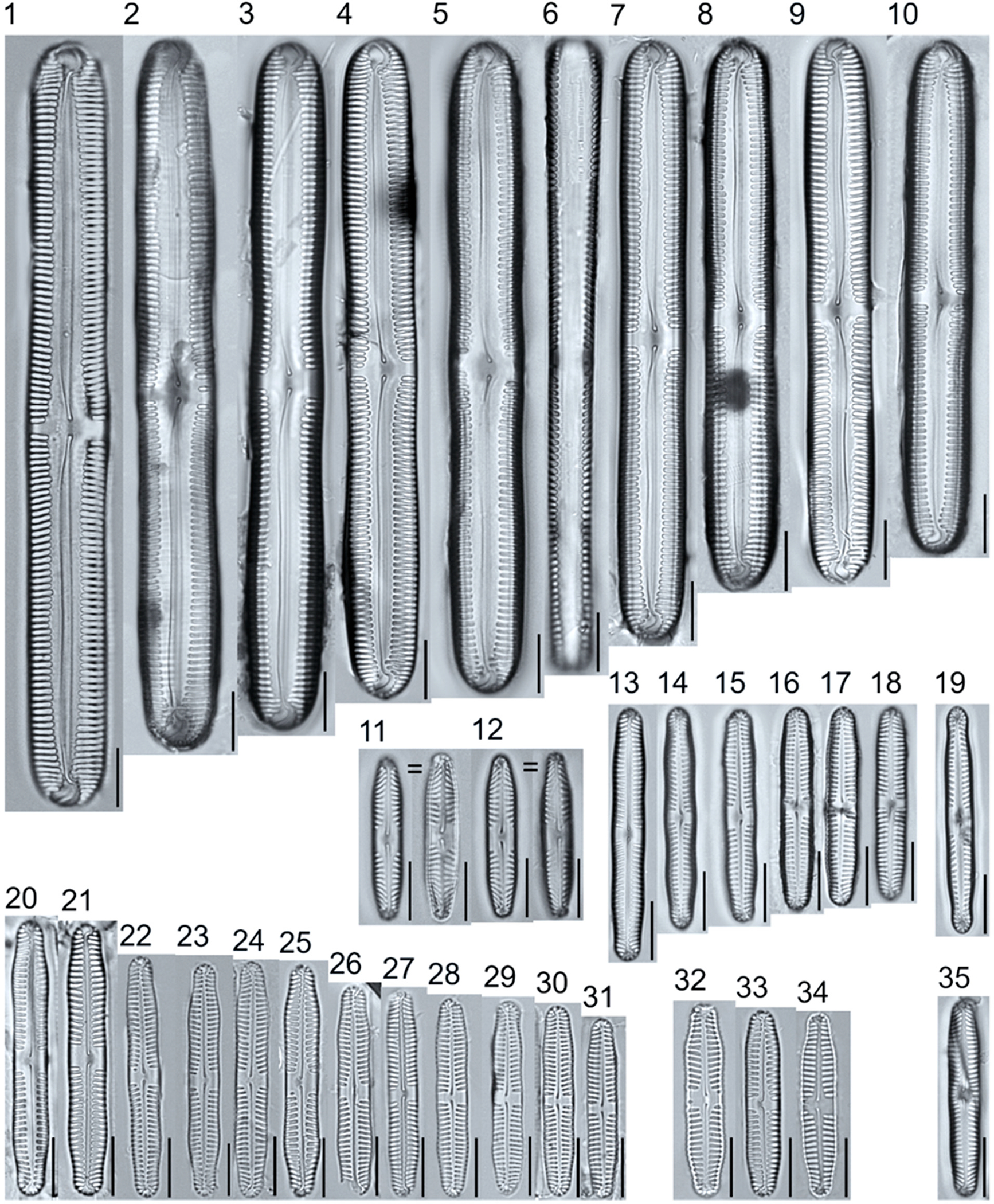
Light micrographs of symmetric biraphid taxa. Figs. 1–10: *Pinnularia crucifera*. Figs. 11–12: *P. obscura*. Figs. 13–18: *P. subcapitata* var. *subrostrata*. Figure 19: *P. subcapitata* var. *elongata*. Figs. 20–31: *P. pulchra*. Figs. 32–34: *P*. cf. *pulchra*. Figure 35: *P. submicrostauron*. Scale bars: 10 μm.

**Plate 15.**
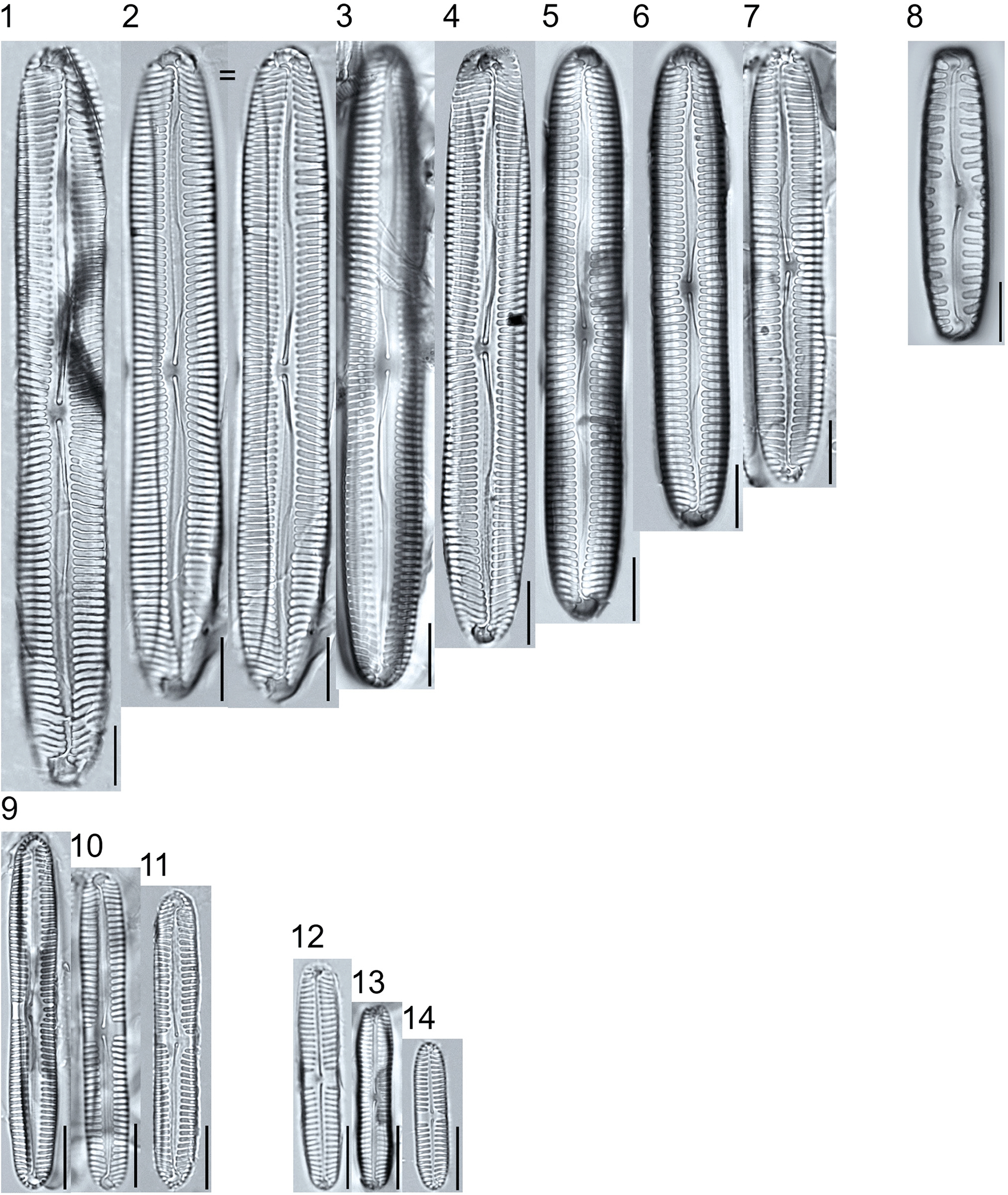
Light micrographs of symmetric biraphid taxa. Figs. 1–7: *Pinnularia viridiformis*. Figure 8: *P. borealis*. Figs. 9–11: *P. ivaloensis*. Figs. 12–14: *Caloneis schroederioides*. Scale bars: 10 μm.

**Plate 16.**
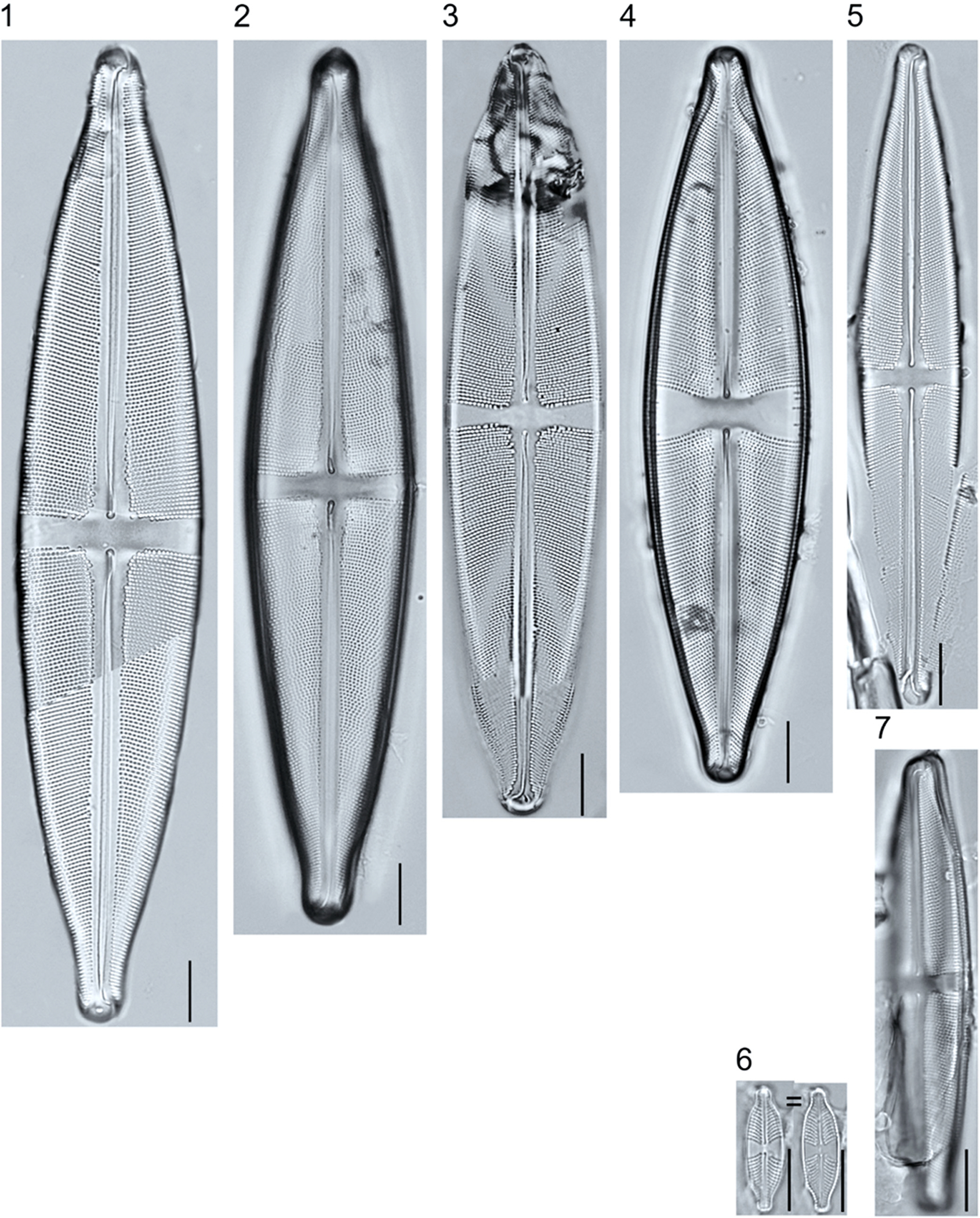
Light micrographs of symmetric biraphid taxa. Figure 1: *Stauroneis heinii*. Figure 2: *S. superkuelbsii*. Figure 3: *S. indianopsis*. Figure 4: *S. sonyae*. Figure 5: *S. subborealis*. Figure 6: *S. borrichii* f. *subcapitata*. Figure 7: *S*. sp.1 APP. Scale bars: 10 μm.

**Plate 17.**
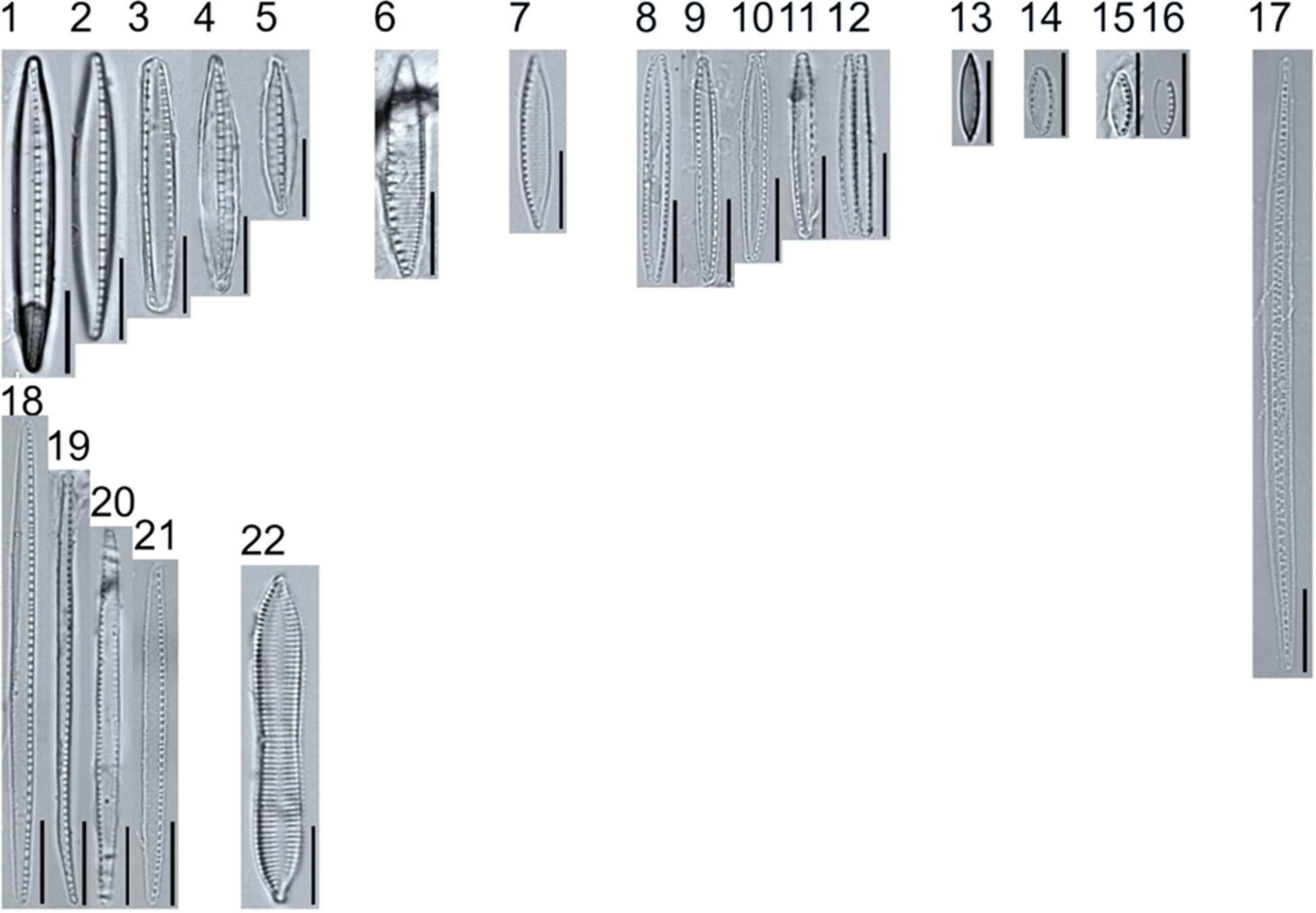
Light micrographs of nitzschioid taxa. Figs. 1–5: *Nitzschia dissipata*. Figure 6: *N. amphibia*. Figure 7: *N. alpina*. Figs. 8–12: *N. palea* var. *debilis*. Figure 13: *N*. cf. *lacuum*. Figure 14: *N. soratensis*. Figs. 15–16: *N. inconspicua*. Figure 17: *N*. cf. *columbiana*. Figs. 18–21: *N. paleacea*. Figure 22: *Tryblionella apiculata*. Scale bars: 10 μm.

**Plate 18.**
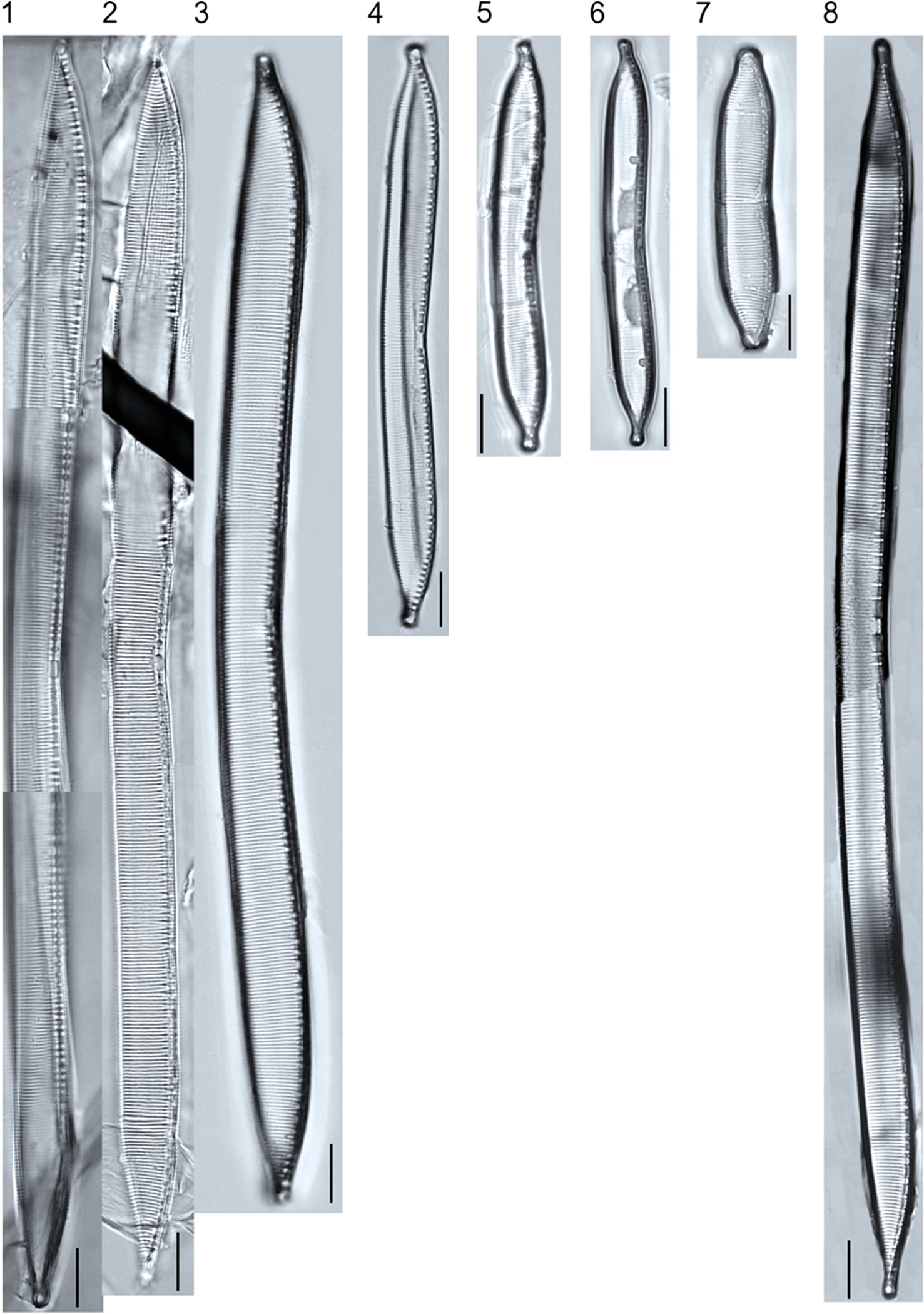
Light micrographs of hantzschioid taxa. Figs. 1–3: *Hantzschia spectabilis*. Figure 4: *H. vivacior*. Figure 5: *H. calcifuga*. Figure 6: *H*. cf. *bardii*. Figure 7: *H*. sp.1 APP. Figure 8: *H. elongata*. Scale bars: 10 μm.

**Table 1.** The overall sampling season (5 sampling dates × 4 replicate plots per fen = 20) mean and ±1 standard deviation (SD) for physiochemical characteristics of the rich, moderate, and poor fen sites. Ranges represent the minimum and maximum of measurements taken across the growing season (May–August 2017).

	Rich			Moderate			Poor		

Characteristic	Mean	SD	Range	Mean	SD	Range	Mean	SD	Range

Water depth (cm)	27.5	7.95	20–35	34.0	11.4	20–46	26.6	8.86	16–34
Water temperature (°C)	18.5	2.29	15–21	18.0	2.88	14–21	15.2	3.53	11–20
Water column pH	6.46	1.29	5.4–8.9	6.42	1.32	5.3–8.5	5.77	1.03	4.8–7.6
DO (mg L^−1^)	3.92	1.95	2.0–7.0	5.71	1.49	4.3–7.8	3.81	2.42	1.0–7.1
Conductivity (μS)	39.0	5.53	31–45	25.4	2.69	24–28	40.8	5.75	34–48
TDN (mg L^−1^)	1.51	0.19	1.3–1.8	1.35	0.26	1.0–1.6	1.65	0.29	1.2–2.0
NO_3_^−^ (μg L^−1^)	7.66	2.14	6.2–8.9	13.2	7.39	7.2–26.0	15.8	9.59	7.4–27
PO_4_^−^ (μg L^−1^)	8.56	3.40	6.4–14	7.54	2.95	4.3–10.7	20.9	6.21	15–28
DOC (mg L^−1^)	32.6	4.32	26–37	29.7	3.14	27–35	62.4	9.41	51–77
PAR (μmol cm^2^ s^−1^)	254.4	246.6	38–448	266.1	225.1	84–672	242.3	207.0	27–484

Note: DO = dissolved oxygen, TDN = total dissolved nitrogen, NO_3−_ = nitrate, PO_4−_ = phosphate, DOC = dissolved organic carbon, PAR = photosynthetically active radiation.

**Table 2.** List of taxa documented in this study, with authorship, dimension range, voucher image references to plates and figures, fen type, accessioned samples at Ball State University, and taxa status in the Red List developed in Germany [25]: 1 = threatened with extinction; 2 = highly threatened; 3 = threatened; G = threat of unknown extent; R = extremely rare; V = near threatened; D = data deficient; * = not threatened; ♦ = not evaluated.

Taxon/Author	Dimensions	Plates and Figure	Fen Type	Sample	[25]
***Achnanthidium alpestre*** (R. L. Lowe and Kociolek) R. L. Lowe and Kociolek in J. R. Johans. et al. 2004	L: 15.0–15.2, W: 3.8	Plate 3, Figures 26 and 27	Poor	25000	♦
***Achnanthidium* cf. *gracillimum*** (F. Meister) Lange-Bert. 2004	L: 8.2–13.6, W: 2.5–3.1	Plate 3, Figures 28–31	Rich; Poor	25002	2
***Achnanthidium minutissimum*** **var. *jackii*** (Rabenh.) Lange-Bert. 1989	L: 11.7, W: 2.4	Plate 3, Figure 32	Poor	25003a	D
***Achnanthidium* sp. 1 APP**	L: 7.6, W:4.1, S: 18–20	Plate 3, Figure 33	Poor	25001	♦
***Amphora copulata*** (Kütz.) Schoeman and R. E. M. Archibald 1986	L: 21.0, W: 5.2, S: 14–15	Plate 4, Figure 4	Moderate	25005a	*
***Amphora ovalis*** (Kütz.) Kütz. 1844	L: 34.3–43.9, W: 8.5–10.8, S: 11–13	Plate 4, Figures 1–3	Rich; Moderate	25006	*
***Amphora pediculus*** (Kütz.) Grunow 1875	L: 6.5–17.1, W:1.5–3.7, S: 13–25	Plate 4, Figures 5–9	Moderate; Poor	25007a	*
***Aulacoseira ambigua*** (Grunow) Simonsen 1979	D: 4.7–7.5,MH: 9.2–11.9, A: 18–20	Plate 1, Figures 6 and 7	Rich; Poor	25008	*
***Caloneis schroederoides*** Foged 1981	L: 23.3–35.8, W: 5.2–6.0, S: 11–15	Plate 15, Figures 12–14	Moderate	25102	♦
***Cocconeis pediculus*** Ehrenb. 1838	L: 20.0–33.2, W: 16.3–23.7, S: 14–20	Plate 3, Figures 1–11	Rich; Moderate; Poor	25009	*
***Cocconeis placentula sensu lato*** Ehrenb. 1838	L: 11.9–23.2, W: 6.6–12.9, S: 16–25	Plate 3, Figures 11–19	Rich; Moderate; Poor	25010	*
***Denticula* cf. *kuetzingii*** Grunow 1862	L: 18.5 S: 16–19 F: 6	Plate 2, Figure 44	Rich	25092b	♦
***Diadesmis* sp. 1APP**	L: 14.7–18.5,W: 5.9–7.4, S: 18–22	Plate 8, Figures 6 and 7	Moderate	25087b	♦
***Diatoma ehrenbergii*** Kütz. 1844	L: 40.9, W: 6.1, C:11–13	Plate 2, Figure 40	Poor	25004b	*
***Diatoma moniliformis*** (Kütz.) D.M.Williams 2012	L: 16.2–25.7, W: 5.5, C: 6–8	Plate 2, Figures 41 and 42	Rich; Poor	25012	*
***Diatoma vulgaris*** Bory 1824	L: 36.8–45.6, W: 11.2–12.8, C: 7–9	Plate 2, Figures 38 and 39	Moderate	25013a	*
***Encyonema groenlandica*** (Foged) Kulikovskiy and Lange-Bert. 2009	L: 28.6–29.6, W: 5.4–5.8, S: 6–8	Plate 5, Figures 34 and 36	Rich; Moderate	25014a	♦
***Encyonema* cf. *groenlandica*** (Foged) Kulikovskiy and Lange-Bert. 2009	L: 18.2–19.3, W: 4.9–5.2, S: 10–12	Plate 5, Figures 38 and 39	Rich; Moderate	25015a	♦
***Encyonema lunatum*** **var. *alaskaense*** (Foged) Metzeltin and Lange-Bert. 2009	L: 36.8–39.7, W: 4.8–5.0, S: 8–10	Plate 5, Figures 30–32	Rich; Moderate	25016a	♦
***Encyonema montana*** Bahls 2017	L: 14.1–17.3, W: 5.5–7.0, S: 12–14	Plate 5, Figures 43–45	Rich; Poor	25017a	♦
***Encyonema neogracile*** Krammer 1997	L: 31.5–44.7, W: 4.7–6.3, S: 11–15	Plate 5, Figures 16–29	Rich; Moderate; Poor	25018a	3
***Encyonema paucistriatum*** (A. Cleve) D. G. Mann 1990	L: 22.1–42.8, W: 5.4–6.5, S: 8–11 dorsal, 12–13 ventral	Plate 5, Figures 1–14	Rich; Moderate; Poor	25019a	2
***Encyonema procerum*** Krammer 1997	L: 31.4, W: 6.8, S: 10–12	Plate 5, Figure 34	Moderate	25015b	1
***Encyonema schimanskii*** Krammer 1997	L: 17.4–20.0, W: 4.5–5.0, S: 14–15	Plate 5, Figures 40–42	Rich; Moderate	25016b	G
***Encyonema silesiacum*** (Bleisch in Rabenh.) D. G. Mann 1990	L: 36.4, W: 8.8, S: 11–12	Plate 5, Figure 33	Moderate	25007b	*
***Encyonopsis cf. microcephala*** (Grunow) Krammer 1997	L: 15.9, W: 3.7, S: 19–20	Plate 6, Figure 65	Rich	25021a	*
***Encyonopsis* cf. *minuta*** Krammer and E. Reichardt 1997	L: 12.3, W: 3.4	Plate 6, Figure 67	Poor	25003b	D
***Encyonopsis montana*** Bahls 2013	L: 43.6, W: 7.1, S: 19–20	Plate 6, Figure 64	Poor	25020	♦
***Encyonopsis thumensis*** Krammer 1997	L: 14.7, W: 3.1, S: 20–22 *	Plate 6, Figure 66	Poor	25004c	G
***Eunotia ambivalens*** Lange-Bert. and Tagliaventi 2011	L: 110.8–154.1, W: 4.8–5.1, S: 12–14c; 15–16a	Plate 21, Figures 6–10	Rich	25022a	G
***Eunotia arcus*** Ehrenberg 1837	L: 27.5–52.9, W: 9.4–10.8, S: 10–12	Plate 24, Figures 19–20	Rich	25023	V
***Eunotia* cf. *bertrandii*** Lange-Bert. and Tagliaventi 2011	L: 15.8–25.6, W: 2.9–3.8, S: 17–20	Plate 22, Figures 28–36	Rich; Moderate; Poor	25056	G
***Eunotia bidens*** Ehrenberg 1843	L: 76.6, W: 13.9, S: 9–10c, 13–14a	Plate 24, Figure 22	Rich	25024	G
***Eunotia bilunaris*** (Ehrenb.) Schaarschm. 1881	L: 35.0–74.1, W: 3.6–4.3, S: 16–19	Plate 23, Figures 7–11	Rich; Moderate	25025	*
***Eunotia curtagrunowii*** Nörpel Schempp and Lange-Bert. 1996	L: 20.0–29.6, W: 7.0–9.8, S: 8–12	Plate 24, Figures 13–18	Rich; Moderate	25026a	G
***Eunotia dorofeyukae*** Lange-Bertalot and Kulikovskiy 2010	L: 40.7, W: 9.4, S: 11–14	Plate 24, Figure 11	Rich	25048	R
***Eunotia ferefalcata*** Kulikovskiy and Lange-Bert. 2011	L: 20.7–55.7 *, W: 3.6–4.3 *, S: 17–21*	Plate 23, Figures 12–16	Rich; Moderate; Poor	25027	♦
***Eunotia flexuosa*** (Brébisson ex Kütz.) Kütz. 1849	L: 147.6–246.7, W: 3.8–4.5, S: 10–13c; 13–15a	Plate 20, Figures 1–6	Moderate; Poor	25028a	3
***Eunotia* cf. *glacialispinosa*** Lange-Bert. and Cantonati 2010	L: 43.9–46.9, W: 4.5–4.6, S: 12–14	Plate 23, Figures 17–19	Rich	25034a	G
***Eunotia cf. groenlandica*** (Grunow) Nörpel-Schempp and Lange-Bert. nom inval. 1996	L: 50.2, W: 5.2, S: 11–12	Plate 23, Figure 20	Moderate	25029	G
***Eunotia* cf. *intermedia*** (Krasske ex Hust.) Nörpel and Lange-Bert. 1993	L: 39.1, W: 3.1, S: 18–19	Plate 25, Figure 38	Moderate	25019b	2
***Eunotia juettnerae*** Lange-Bert. 2011	L: 108.6, W: 4.0, S: 15–17	Plate 23, Figure 1	Poor	25031	G
***Eunotia cf. julma*** Lange-Bert. 2011	L: 155.8, W: 4.5, S: 15–16	Plate 21, Figure 8	Poor	25032	♦
***Eunotia julma*** Lange-Bert. 2011	L: 93.0–219.2, W: 3.8–5.0, S: 13–18	Plate 21, Figures 1–7	Moderate; Poor	25033	♦
***Eunotia krammeri*** Kulikovskiy, Lange-Bertalot, Genkal, and Witkowski 2010	L: 52.5–58.8, W: 2.4–2.5, S: 18–20	Plate 23, Figures 2–3	Poor	25031	♦
***Eunotia cf. major*** (W. Smith) Rabenh. 1864	L: ~55.1, W: 13.5, S: 11–15	Plate 25, Figure 28	Moderate	25035	2
***Eunotia cf. mucophila*** (Lange-Bert., Nörpel-Schempp, and Alles) Lange-Bert. 2007	L: 59.3–68.5, W: 3.2–3.2, S: 21–23	Plate 20, Figures 12 and 13	Poor	25036a	G
***Eunotia mucophila*** (Lange-Bert., Nörpel-Schempp, and Alles) Lange-Bert. 2007	L: 16.1–47.1, W: 2.7–3.1, S: 18–22	Plate 20, Figures 14–22	Poor	25037a	G
***Eunotia naegelii*** Mig. 1907	L: 103.8–123.1, W: 2.7–3.1, S: 15–17c; 17–20a	Plate 22, Figures 1–5	Poor	25038	3
***Eunotia neocompacta*** Mayama 1998	L: 20.4–60.1, W: 3.8–5.2, S: 17–18	Plate 25, Figures 1–13	Moderate; Poor	25039	2
***Eunotia neocompacta*** **var. *vixcompacta*** Lange-Bert. 2011	L: 22.6–30.8, W: 3.9–4.7, S: 17–18	Plate 25, Figures 14–17	Moderate; Poor	25040a	G
***Eunotia paludosa*** Grunow 1862	L: 30.1–48.9, W: 3.0–3.5, S: 18–20	Plate 25, Figures 29–36	Moderate; Poor	25041	V
***Eunotia* cf. *paludosa*** Grunow 1862	L: 24.8–31.3, W: 2.9–3.2, S: 17–20	Plate 22, Figures 45 and 46	Rich; Moderate	25030	V
***Eunotia panda*** J. Veselá and J. R. Johans 2014	L: 38.7–81.1, W: 3.8–4.0, S: 14–17	Plate 23, Figures 4–6	Moderate; Poor	25042	♦
***Eunotia praerupta*** Ehrenberg 1843	L: 37.4–65.6, W: 13.6–14.8, S: 6–9	Plate 24, Figures 1–6	Moderate; Poor	25043	2
***Eunotia praerupta* var. *inflata*** (Grunow) Freng. 1924	L: 32.2–58.3, W: 13.7–16.1, S: 7–12	Plate 24, Figures 7–10	Moderate; Poor	25044	♦
***Eunotia pseudoflexuosa*** Hustedt 1949	L: 97.9–114.6, W: 3.9–5.7, S: 13–15	Plate 20, Figures 7–9	Moderate	25045	♦
***Eunotia pseudogroenlandica*** Lange-Bert. and Tagliaventi 2011	L: 17.5–34.4, W: 2.8–3.3, S: 16–18	Plate 22, Figures 37–44	Poor	25046	G
***Eunotia pseudoparallela*** A. Cleve 1934	L: 64.9–117.9, W: 8.8–11.3, S: 12–15	Plate 19, Figures 1–13	Moderate; Poor	25047	♦
***Eunotia cf. rushforthii*** Furey, R. L. Lowe, and Johansen 2011	L: 10.7–14.8, W: 2.4–2.8, S: 20–25	Plate 25, Figures 39–42	Moderate; Poor	25049a	♦
***Eunotia* cf. *scandiorussica*** Kulikovskiy, Lange-Bert.,	L: 19.0, W: 5.2, S: 16–17	Plate 23, Figure 22	Moderate	25050	♦
***Eunotia scandiorussica*** Kulikovskiy, Lange-Bertalot, Genkal, and Witkowski 2010	L: 9.0–34.8, W: 4.6–5.9, S: 13–19	Plate 22, Figures 11–27	Rich; Moderate; Poor	25051a	♦
***Eunotia sedina*** Lange-Bert., Bąk, and Witkowski 2011	L: 28.2–50.3, W: 5.3–7.1, S: 10–16c, 17–18a	Plate 25, Figures 19–27	Rich; Moderate	25052a	G
***Eunotia septentrionalis*** Østrup 1897	L: 23.7–24.3, W: 6.5–6.8, S: 13–14	Plate 24, Figures 23–24	Poor	25028b	♦
***Eunotia* cf. *silesioscandica*** Lange-Bert. and Sienkiewicz 2011	L: 36.4, W: 5.4, S: 16–17	Plate 23, Figure 21	Rich	25052b	♦
***Eunotia subcapitata*** Kulikovskiy, Lange-Bert., Genkal, and Witkowski 2010	L: 86.0–98.9, W: 3.1–3.6, S: 15–20	Plate 22, Figures 6–10	Rich; Moderate; Poor	25053	♦
***Eunotia superbidens*** Lange-Bert. 2011	L: 69.5, W: 14.7, S: 10–11	Plate 24, Figure 21	Moderate	25054	G
***Eunotia superpaludosa*** Lange-Bert. 2011	L: 40.5, W: 4.9, S: 19–20	Plate 25, Figure 18	Poor	25055	1
***Eunotia* cf. *trinacria*** Krasske 1929	L: 39.7, W: 3.7, S: 17–18	Plate 25, Figure 37	Poor	25057	3
***Eunotia ursamaioris*** Lange-Bert. and Nörpel-Schempp 1999	L: 16.0–39.2, W: 4.3–5.6, S: 14–16c; 17–18a	Plate 21, Figures 12–23	Poor	25058	G
***Eunotia valida*** Hust. 1930	L: 70.0–107.5, W: 5.6–5.8, S: 13–14	Plate 20, Figures 10 and 11	Rich; Moderate	25059	G
***Eunotia* sp. 1 APP (teratology)**	L: 17.8, W: 5.6, S: 16–18	Plate 24, Figure 12	Poor	25060	♦
***Eunotia* sp. 2 APP**	L: 67.2, W: 7.0, S: 13–14	Plate 23, Figure 23	Moderate	25061	♦
***Eunotia* sp. 3 APP**	L: 18.3, W: 3.1, S: 18–19	Plate 25, Figure 43	Poor	25062	♦
***Eunotia* sp. 4 APP**	L: 10.1–22.1, W: 2.9–5.4, S: 14–20	Plate 25, Figures 44 and 45	Poor	25051b	♦
***Fragilaria rumpens*** (Kütz.) G. W. F. Carlson 1913	L: 26.1, W: 4.3, S: 18–19	Plate 2, Figure 45	Moderate	25013b	*
***Gomphoneis herculeana*** (Ehrenb.) Cleve 1894	L: 87.4, W: 25.5, S: 11–14	Plate 6, Figure 1	Poor	25063	♦
***Gomphonema barrowiana*** R. M. Patrick and Freese 1961	L: 30.4, W: 4.4, S: 16–17	Plate 5, Figure 65	Rich	25064	♦
***Gomphonema brebissonii*** Kütz. 1849	L: 32.1–51.2, W: 5.3–7.6, S: 9–13	Plate 6, Figures 54–63	Rich; Moderate	25065	*
***Gomphonema* cf. *clavatulum*** E. Reichardt 1999	L: 17.6, W: 4.1, S: 14–16	Plate 5, Figure 54	Rich	25021b	*
***Gomphonema* cf. *consector*** Hohn and Hellerman 1963	L: 15.4–20.3, W: 3.8–4.3, S: 13–16	Plate 5, Figures 63 and 64	Rich	25066a	♦
***Gomphonema* cf. *frigidum*** (Lange-Bert.) Lange-Bert. and Reichardt in Lange-Bert. and Genkal 1999	L: 18.0–21.8, W: 3.5–4.2, S: 12–16	Plate 5, Figures 55–58	Rich; Moderate	25067	♦
***Gomphonema* cf. *himalayaense*** (Jüttner) Jüttner et al., 2018	L: 17.1, W: 5.7, S: 12–13	Plate 5, Figure 67	Moderate	25013c	♦
***Gomphonema italicum*** Kütz. 1844	L: 26.4, W: 10.8, S: 11–12	Plate 5, Figure 69	Moderate	25040b	*
***Gomphonema lagerheimii*** A. Cleve 1895	L: 33.3–55.5, W: 4.3–7.0, S: 12–18	Plate 6, Figures 28–46	Rich; Moderate	25068	2
***Gomphonema lateripunctatum*** E. Reichardt and Lange-Bert. 1991	L: 51.5, W: 5.2, S: 12–14	Plate 6, Figure 14	Rich	25069	V
***Gomphonema montanum*** **var. *minutum*** (Skvortzow) Z. X. Shi 2014	L: 18.0–21.5, W: 3.8–3.9, S: 9–12	Plate 5, Figures 61 and 62	Rich	25014b	♦
***Gomphonema olivaceum*** **var. *densestriatum*** Foged 1982	L: 16.2, W: 6.2, S: 12–14	Plate 5, Figure 68	Poor	25003c	♦
***Gomphonema* cf. *parvulum*** (Kütz.) Kütz. 1849	L: 31.4–32.7, W: 6.0–6.9, S: 13–14	Plate 6, Figures 12 and 13	Rich	25070a	*
***Gomphonema parvulum*** (Kütz.) Kütz. 1849	L: 21.7–25.0, W: 6.3–8.5, S: 15–20	Plate 6, Figures 48–50	Rich; Moderate	25071	*
***Gomphonema parvulum*** **f. *saprophilum*** Lange-Bert. and E. Reichardt 1993	L: 18.0, W: 6.9, S: 14–15	Plate 5, Figure 66	Moderate	25005b	♦
***Gomphonema* cf. *parapygmaeum*** (Jüttner) Jüttner and Kociolek 2018	L: 18.5–23.2, W: 3.6–4.1, S: 11–14	Plate 5, Figures 59 and 60	Rich; Poor	25028c	♦
***Gomphonema* cf. *raraense*** Jüttner and S. Gurung 2018	L: 21.9–29.2, W: 3.4–4.3, S: 12–15	Plate 6, Figures 15–27	Rich; Moderate	25072	♦
***Gomphonema* sp. 1 APP**	L: 64.5–74.5, W: 8.5–8.9, S: 10–12	Plate 6, Figures 2–4	Rich	25073	♦
***Gomphonema* sp. 2 APP**	L: 55.8, W: 6.6, S: 15–16	Plate 6, Figure 5	Moderate	25018b	♦
***Gomphonema* sp. 3 APP**	L: 38.2–38.2, W: 5.1–5.2, S: 14–16	Plate 6, Figures 6–8	Moderate	25074a	♦
***Gomphonema* sp. 4 APP**	L: 30.5–34.1, W: 5.1–5.4, S: 17–19	Plate 6, Figures 9–11	Moderate	25075a	♦
***Gomphonema* sp. 5 APP**	L: 46.4, W: 5.7, S: 11–13	Plate 6, Figure 5	Rich	25076	♦
***Gomphonema* sp. 6 APP**	L: 36.5, W: 5.4, S: 15–16	Plate 6, Figure 51	Rich	25017b	♦
***Gomphonema* sp. 7 APP**	L: 38.7, W: 5.6, S: 13–14	Plate 6, Figure 52	Rich	25077a	♦
***Gomphonema* sp. 8 APP**	L: 40.9, W: 7.3, S: 13–14	Plate 6, Figure 53	Rich	25021c	♦
***Hantzschia* cf. *bardii*** Lange-Bert., Cavacini, Tagliaventi, and Alfinito 2003	L: 69.5, W: 7.3, S: 24–25 F: 4–6	Plate 18, Figure 6	Rich	25078a	♦
***Hantzschia calcifuga*** E. Reichardt and Lange-Bert. 2004	L: 70.2, W: 7.5, S: 18–20	Plate 18, Figure 5	Rich	25070b	D
***Hantzschia elongata*** (Hantzsch) Grunow 1877	L: 213.3, W: 9.5, S: 17–18	Plate 18, Figure 8	Rich	25079	G
***Hantzschia spectabilis*** (Ehrenb.) Hust. 1959	L: 196.4–215.6, W: 9.5–10.7, S: 15–18	Plate 18, Figures 1–3	Rich	25080	*
***Hantzschia vivacior*** Lange-Bert. 1993	L: 105.9, W: 8.5, S: 17–18, F: 6–7	Plate 18, Figure 4	Rich	25081	D
***Hantzschia* sp. 1 APP**	L: 54.7, W: 10.9, S: 18–19, F: 4–6	Plate 18, Figure 7	Rich	25022b	♦
***Hippodonta pseudopinnularia*** Lange-Bert. 2001	L: 17.9, W: 4.6, S: 9–10	Plate 9, Figure 11	Poor	25036b	♦
***Kobayasiella parasubtilissima*** (H. Kobayasi and T. Nagumo) Lange-Bert. 1999	L: 29.8–34.6, W: 4.1–4.8	Plate 9, Figures 1–10	Moderate; Poor	25082	V
***Lindavia ocellata*** (Pantocsek) Nakov et al. 2015	D: 6.2–15.6, S/C: 16.3–25.0	Plate 1, Figures 8–19	Moderate	25083	*
***Melosira varians*** C.Agardh 1827	D: 14.2–22.3	Plate 1, Figures 1–4	Rich	25084a	*
***Microcostatus* sp. 1 APP**	L: 9.9, W: 3.9, S: 24–25	Plate 9, Figure 13	Poor	25004a	♦
***Navicula antonii*** Lange-Bert. 2000	L: 19.1–20.1, W: 6.9–7.5, S: 11–14	Plate 8, Figures 15 and 16	Rich	25066b	*
***Navicula* cf. *catalanogermanica*** Lange-Bert. and G. Hofmann 1993	L: 15.1, W: 7.5, S: 12–12	Plate 8, Figure 10	Rich	25034b	*
***Navicula caterva*** Hohn and Hellermann 1963	L: 15.2, W: 5.2, S: 18–19	Plate 8, Figure 11	Rich	25034c	R
***Navicula* cf. *cincta*** (Ehrenb.) Ralfs 1861	L: 16.3, W: 4.0, S: 9–10	Plate 9, Figure 12	Poor	25036c	*
***Navicula cryptotenella*** Lange-Bert. 1985 S: 14–15	L: 23.7, W: 5.1,	Plate 8, Figure 8	Poor	25028d	*
***Navicula erifuga*** Lange-Bert. 1985	L: 25.1, W: 5.9, S: 14–14	Plate 8, Figure 14	Poor	25028e	*
***Navicula germainii*** J. H. Wallace 1960	L: 37.4, W: 8.1, S: 14–15	Plate 8, Figure 2	Rich	25084b	*
***Navicula gregaria*** Donkin 1861	L: 22.6–29.1, W: 6.3–6.8, S: 15–19	Plate 8, Figures 3–5	Rich; Poor	250120	*
***Navicula* cf. *streckerae*** Lange-Bert. and Witkowski 2000	L: 31.3, W: 8.0, S: 9–10	Plate 8, Figure 13	Poor	25086	*
***Navicula tenelloides*** Hust. 1937	L: 20.1, W: 4.0, S: 15–17	Plate 8, Figure 17	Rich	25085c	*
***Navicula tripunctata*** (O. F. Müller) Bory 1822	L: 33.8–44.1, W: 7.8–8.8, S: 10–12	Plate 8, Figures 1 and 12	Rich, Moderate	25085a	*
***Navicula metareichardtiana*** Lange-Bert. and Kusber 2019	L: 17.2, W: 4.9, S: 14–15	Plate 8, Figure 9	Moderate	25005c	*
***Neidium bisulcatum*** (Lagerstedt) Cleve 1894	L: 28.6–69.3, W: 5.6–9.6	Plate 9, Figures 14–23	Moderate; Poor	25088	3
***Neidium bisulcatum*** **var. *subampliatum*** Krammer 1985	L: 36.6, W: 8.5, S: 22–24	Plate 9, Figure 24	Poor	25089a	3
***Nitzschia alpina*** Hust. 1943	L: 22.4, W: 3.9, S: 20–21, F: 11–12	Plate 17, Figure 7	Rich	25085d	3
***Nitzschia amphibia*** Grunow 1862	L: 26.8, W: 5.2, S: 16–17, F: 10	Plate 17, Figure 6	Moderate	25087c	*
***Nitzschia* cf. *columbiana*** Sovereign 1960	L: 74.1, W: 3.5, F: 9	Plate 17, Figure 17	Rich	25014c	♦
***Nitzschia dissipata*** (Kütz.) Rabenh. 1860	L: 20.2–38.4, W: 4.5–5.4, F: 8–10	Plate 17, Figures 1–5	Rich	25034d	*
***Nitzschia inconspicua*** Grunow 1862	L: 7.0–7.8, W: 2.6–2.7, F: 14	Plate 17, Figures 15–16	Moderate	25090	♦
***Nitzschia* cf. *lacuum*** Lange-Bert. 1980	L: 10.9, W: 2.4	Plate 17, Figure 13	Rich	25034e	G
***Nitzschia palea*** **var. *debilis*** (Kütz.) Grunow 1880	L: 21.9–27.6, W: 1.9–3.6, S: 28–30, F: 12–15	Plate 17, Figures 8–12	Rich; Moderate; Poor	25091	*
***Nitzschia paleacea*** (Grunow) Grunow in Van Heurck 1881	L: 41.0–58.2, W: 1.8–3.0, F: 11–14	Plate 17, Figures 18–21	Rich; Moderate	25016c	*
***Nitzschia soratensis*** E. A. Morales and M. L. Vis 2007	L: 8.4, W: 3.0, F: 12	Plate 17, Figure 14	Poor	25001b	*
***Odontidium hyemale*** (Roth) Kütz. 1844	L: 31.9, W: 8.3, S: 26–28 C: 4–6	Plate 2, Figure 43	Poor	25092a	♦
***Pinnularia aequilateralis*** R. M. Patrick and Freese 1961	L: 71.3–73.1, W: 8.6, S: 8–10	Plate 13, Figures 18–19	Moderate	25026b	♦
***Pinnularia aestaurii*** ***var. interrupta*** (Hustedt) A. Cleve 1934	L: 68.9–82.5, W: 9.8–11.9, S: 7–9c, 9–10a	Plate 13, Figures 1–17	Rich; Moderate	25093a	♦
***Pinnularia borealis*** Ehrenb. 1843	L: 48.9, W: 11.5, S: 4–5	Plate 15, Figure 8	Rich	25094	*
***Pinnularia crucifera*** A. Cleve 1934	L: 86.8–129.1, W: 10.8–14.6, S: 8–10	Plate 14, Figures 1–11	Rich; Moderate; Poor	25095	♦
***Pinnularia cruxarea*** Krammer 2000	L: 78.6, W: 11.0, S: 9–10	Plate 12, Figure 12	Rich	25078b	R
***Pinnularia genkalii*** Krammer and Lange-Bert. 2000	L: 121.4–131.1, W: 16.3–19.5, S: 5–8	Plate 11, Figures 1–3	Moderate	25096	♦
***Pinnularia moelderi*** D. M. Williams, Bing Liu, and Taxböck 2022	L: 84.4–97.2, W: 10.8–12.6, S: 8–9	Plate 12, Figures 1–7	Rich; Moderate	25097	♦
***Pinnularia ilkaschoenfelderae*** Krammer nom. inval. 2000	L: 87.6–110.6, W: 13.2–15.8, S: 5–6c, 6–8a	Plate 11, Figures 4–5	Rich; Moderate	25026c	♦
***Pinnularia_ivaloensis*** Krammer 2000	L: 46.9–55.6, W: 6.6–6.8, S: 9–12	Plate 15, Figures 9–11	Rich	25098	♦
***Pinnularia neomajor*** Krammer 1992	L: 118.5–181.3, W: 17.0–21.0, S: 6–8	Plate 10, Figures 1–6	Rich; Moderate	25099	G
***Pinnularia obscura*** Krasske 1932	L: 27.2–28.0, W: 5.2–5.5, S: 11–13	Plate 14, Figures 11–12	Rich; Moderate	25100a	*
***Pinnularia pulchra*** Østrup 1897	L: 30.3–46.6, W: 5.2–6.6, S: 9–13	Plate 14, Figures 20–31	Rich; Moderate; Poor	25101	♦
***Pinnularia* cf. *pulchra*** 0strup 1897	L: 31.1–32.8, W: 5.9–6.2, S: 10–12	Plate 14, Figures 32–34	Rich; Moderate; Poor	25103	♦
***Pinnularia spitsbergensis*** Cleve 1895	L: 81.5, W: 9.7, S: 14–14	Plate 12, Figure 13	Rich	25093b	♦
***Pinnularia subcapitata*** **var. *elongata*** Krammer 1992	L: 38.4, W: 4.9, S: 10–11	Plate 14, Figure 19	Rich	25104	*
***Pinnularia subcapitata*** **var. *subrostrata*** Krammer 1992	L: 32.6–42.7, W: 5.3–5.7, S: 12–14	Plate 14, Figures 1–18	Moderate	25105	D
***Pinnularia submicrostauron*** S. Schroeter in Krammer 1992	L: 33.1, W: 5.4, S: 12–13	Plate 14, Figure 35	Moderate	25037b	D
***Pinnularia abaujensis*** **var. *subundulata*** (Ant.Mayer) R. M. Patrick 1966	L: 50.4–69.9, W: 6.5–8.0, S: 7–13	Plate 12, Figures 8–10	Rich; Moderate	25106	♦
***Pinnularia viridiformis* var. *minor*** Krammer 2000	L: 76.0–124.0, W: 11.8–14.8, S: 7–9	Plate 15, Figures 1–7	Rich; Moderate	25107	D
***Pinnularia* sp.1 APP**	L: 71.6, W: 9.8, S: 9–10	Plate 12, Figure 11	Rich	25077b	♦
***Pinnularia* sp. 2 APP**	L: 58.8–100.8, W: 7.5–11.7, S: 7–10	Plate 12, Figures 14–19	Rich; Moderate	25108	♦
***Planothidium frequentissimum*** (Lange-Bert.) Lange-Bert. 1999	L: 7.6–14.4, W: 4.0–5.1, S: 15–18	Plate 3, Figures 22–24	Moderate	25040c	*
***Planothidium rostratoholarcticum*** Lange-Bertalot and Bąk 2015	L: 9.6, W: 5.3, S: 13–15	Plate 3, Figure 25	Moderate	25100b	*
***Psammothidium* cf. *microscopicum*** (Cholnoky) S. Blanco 2016	L: 8.8, W: 3.9, S: 20–24	Plate 3, Figure 35	Rich	25034e	D
***Reimeria sinuata*** (W. Greg.) Kociolek and Stoermer 1987	L: 14.2, W: 4.0, S: 12–14	Plate 5, Figure 53	Poor	25028f	*
***Rhoicosphenia abbreviata*** (C. Agardh) Lange-Bert. 1980	L: 17.2–26.3, W: 5.0–5.2, S: 10–13	Plate 5, Figures 48–52	Moderate; Poor	25109a	*
***Rhoicosphenia* cf. *stoermeri*** E. W. Thomas and Kociolek 2015	L: 33.3, S: 10–11	Plate 5, Figure 47	Rich	25110	♦
***Rossithidium petersenii*** (Hustedt) Round and Bukhtiyarova 1996	L: 15.0, W: 4.1	Plate 3, Figure 34	Poor	25028g	♦
***Skabitschewskia oestrupii*** (A. Cleve) Kulikovskiy and Lange-Bert. 2015	L: 15.9, W: 8.5, S: 14–18	Plate 3, Figures 20–21	Poor	25089b	V
***Stauroneis borrichii f. subcapitata*** (J. B. Petersen) Hust. 1957	L: 20.1 gm, W: 6.0 gm, S: 17–18	Plate 16, Figure 6	Moderate	25026d	♦
***Stauroneis heinii*** Lange-Bert. and Krammer 1999	L: 157.6, W: 30.2, S: 13–16	Plate 16, Figure 1	Moderate	25111	♦
***Stauroneis indianopsis*** Bahls 2010	L: 124, W: 24.8, S: 17–17	Plate 16, Figure 3	Moderate	25112	♦
***Stauroneis sonyae*** Kulikovskiy, Lange-Bert., Witkowski, and Dorofeyuk 2010	L: 118.8, W: 27.0, S: 16–17	Plate 16, Figure 4	Poor	25001c	♦
***Stauroneis subborealis*** Bahls 2010	L: 106.6, W: 16.8, S: 18–19	Plate 16, Figure 5	Moderate	25026e	♦
***Stauroneis superkuelbsii*** Bahls 2010	L: 141.2, W: 26.3, S: 18–20	Plate 16, Figure 2	Moderate	25100c	♦
***Stauroneis* sp. 1 APP**	L: 73.2, W: ~13.1, S: 19–20	Plate 16, Figure 7	Moderate	25075b	♦
***Staurosira* cf. *construens*** Ehrenb. 1843	L: 7.8, W: 4.6, S: 15–16	Plate 2, Figure 52	Moderate; Poor	25074b	*
***Staurosira construens var. venter*** (Ehrenb.) P. B. Ham. 1992	L: 5.7, W: 4.5, S: 12–14	Plate 2, Figure 51	Rich	25114	♦
***Staurosirella leptostauron*** (Ehrenb.) D. M. Williams and Round 1988	L: 13.8–15.5, W: 9.3–10.4, S: 5–11	Plate 2, Figures 46–47	Moderate; Poor	25113	♦
***Staurosirella pinnata*** (Ehrenb.) D. M. Williams and Round 1987	L: 7.9–10.7, W: 5.1–5.3, S: 5–9	Plate 2, Figures 48–50	Moderate	25109b	♦
***Stenopterobia anceps*** (F. W. Lewis) Bréb. ex Van Heurck 1896	L: ~85.7–150.2, W: 5.7–7.4, S: 14–22	Plate 7, Figures 1 and 2	Rich	25016c	♦
***Stenopterobia delicatissima*** (F. W. Lewis) Bréb. ex Van Heurck 1896	L: 53.2–76.0, W: 4.0–4.6, S: 18–28, F: 4–7	Plate 7, Figures 3–12	Poor	25115	2
***Stephanocyclus meneghinianus*** (Kütz.) Kulikovskiy, Genkal, and Kociolek 2022	D: 11.9, S/C: 6.5	Plate 1, Figure 5	Moderate	25011	*
***Tabellaria fenestrata*** (Lyngbye) Kütz. 1844	L: 23.6–49.0, W: 4.0–5.4, S: 14–20	Plate 2, Figures 1–15	Rich; Moderate; Poor	25116	V
***Tabellaria flocculosa*** (Roth) Kütz. 1844	L: 17.0–52.7, W: 4.5–6.7, S: 13–19	Plate 2, Figures 19–28	Rich; Moderate; Poor	25117	*
***Tabellaria* sp. 1 APP**	L: 67.8–88.9, W: 6.6–8.2, S: 13–14 c; 15–20 a	Plate 2, Figures 16–18	Moderate	25118	♦
***Tabellaria* sp. 2 APP**	L: 71.6–93.1, W: 3.6–5.1, S: 12–18	Plate 2, Figures 29–37	Rich; Moderate	25119	♦
***Tryblionella apiculata*** W.Greg. 1857	L: 42.1, W: 5.3, S: 14–15	Plate 17, Figure 22	Rich	25085e	♦

**Dimension notes:** All length and width units are μm. L = valve length, W = valve width, S = stria number in 10 μm (“c” being measured at central area; “a” being measured at apices), MH = mantle height, A = areolae in 10 μm, F = fibulae in 10 μm, S/C = striae by circumference in 10 μm, D = diameter, C = costa number in 10 μm and ~ = best estimation for a broken or distorted valve.

## Data Availability

No new data were created for this project.
