# Supplementary material for: A Voucher Flora of Diatoms from Fens in the Tanana River Floodplain, Alaska
Source: Water (Basel). Author manuscript; Available in PMC 2024 Aug 2. (PMC10750759; doi:10.3390/w15152803)
Supplement: Supplement1 [file NIHMS1948592-supplement-Supplement1.zip › File S1-taxonomic authority references.pdf]

Taxonomic references:

Agardh, C.A. *Systema Algarum Adumbravit.* C.A. Agardh, Lundae: Literis Berlingianis, Lundae, 1824; pp. 1–312.

Bahls, L.L. (2010). *Northwestern Diatoms* Stauroneis *in the northern Rockies: 50 species of* Stauroneis sensu stricto *from western Montana, northern Idaho, northeastern Washington and southwestern Alberta, including 16 species described as new*. pp. [1]-172. Helena, Montana: Montana Diatom Collection.

Bąk, M & Lange-Bertalot, H. (2015 '2014'). Four small-celled *Planothidium* species from Central Europe proposed as new to science. *Oceanological and Hydrobiological Studies* 43(4): 346-359, 11 figs.

Blanco, S. (2016). A nomenclatural note on two species of the Achnanthidiaceae (Bacillariophyta).. *Notulae algarum* 4: 1-2, no figs.

Bory de Saint-Vincent, J.B.M.; coll. *Dictionnaire Classique d'Histoire Naturelle Paris*. Vol 2; Rey et Gravier, libraireséditeurs; Baudouin Frères, libraires-éditeurs. Paris, France, 1822; pp. 1–604. https://doi.org/10.5962/bhl.title.33901

Bory de Saint-Vincent, J.B.M.; coll. *Dictionnaire Classique d'Histoire Naturelle Paris*, Vol. 5.; Rey et Gravier, libraireséditeurs; Baudouin Frères, libraires-éditeurs. Paris, France, 1824; pp. 1–653.

Bory de Saint-Vincent, J.B.M.; coll. *Dictionnaire Classique d'Histoire Naturelle Paris*. Vol 9; Rey et Gravier, libraireséditeurs; Baudouin Frères, libraires-éditeurs. Paris, France, 1826; pp. 1–596. https://doi.org/10.5962/bhl.title.33901

Bory de Saint-Vincent, J.B.G.M. (1822-1831). *Dictionnaire classique d'histoire naturelle* par Messieurs Audouin, Isid. Bourdon, Ad. Brongniart, De Candolle, Daudebard de Férusac, A. Desmoulins, Drapiez, Edwards, Flourens, Geoffroy de Saint-Hilaire, A. De Jussieu, Kunth, G. de Lafosse, Lamouroux, Latreille, Lucas fils, Presle-Duplessis, C. Prévost, A. Richard, Thiébaut de Berneaud, et Bory de Saint-Vincent. Ouvrage dirigé par ce dernier collaborateur, et dans lequel on a ajouté, pour le porter au niveau de la science, un grand nombre de mots qui n'avaient pu faire partie de la plupart des Dictionnaires antérieurs. pp. 17 vols. Paris: Rey et Gravier; Baudoin frères.

Brébisson, A. de. *Considérations sur les Diatomées et essai d'une classification des genres et des espèces appartenant à cette famille*. Brée l'Ainé Imprimeur-Libraire: Falaise, France, 1838; pp. 1–22.

Carlson, G.W.F. (1913). Süsswasser-Algen aus der Antarktis, Süd-Georgien und den Falkland Inseln. *Wissenschaftliche Ergebnisse der Schwedischen Südpolar-Expedition 1901-1903, unter leitung von Dr. Otto Nordenskjöld. Stockholm, Lithographisches Institut des Generalstabs* 4(14): 1-94, 3 pls.

Cleve, P.T. (1891) The Diatoms of Finland. *Acta Soc. Fauna Flora Fenn* **1891***, 8*(2): 1–68. https://doi.org/10.5962/bhl.title.64355

Cleve, P.T. (1894). Synopsis of the naviculoid diatoms. Part I. *Kongliga Svenska Vetenskapsakademiens Handlingar Series 4* 26(2): 1-194, 5 pls.

Cleve, P.T. (1895). Synopsis of the naviculoid diatoms. Part II. *Kongliga Svenska Vetenskapsak Akademiens Handlingar* 27(3): 1-219, 4 pls.

Cleve-Euler, A. (Cleve). The Diatoms of Finnish Lapland. *Societas Scientiarum Fennica. Commentationes Biologicae.,* **1934***, 4*(14): 1–154.

Cleve-Euler, A. *Die Diatomeen von Schweden und Finnland. Teil IV. Biraphideae 2.* Kongliga Svenska Vetenskaps-Akademiens Handligar, Fjärde Serien, Band 5, Nr. 4.; Almqvist & Wiksells Boktryckeri: Stockholm, Sweden, 1955; pp. 1–232.

De Toni, G.B.; Forti, A. Contributo alla conoscenza del plancton del Lago Vetter. *Atti del R. Istituto veneto di scienze, lettere ed arti* **1900***, 59*(2), 537–568.

Donkin, A.S. (1861). On the marine Diatomaceae of Northumberland with a description of several new species. *Quarterly Journal of Microscopical Science, New Series* 1: 1-15, pl. I.

Ehrenberg, C.G. Über die Entwicklung und Lebensdauer der Infusionsthiere; nebst ferneren Beiträgen zu einer Vergleichung ihrer organischen Systeme. *Abh. Konigl. Akad. Wiss. Berlin 1831* 1832, 1–154.

Ehrenberg, C.G. Zusätze zur Erkenntniss grosser organischer Ausbildung in den kleinsten thierischen Organismen. *Abhandlungen der Königlichen Akademie der Wissenschaften zu Berlin* 1836: 150–181, 1 pl.

Ehrenberg, C.G. Über ein aus fossilen Infusorien bestehendes, 1832 zu Brod verbacknes Bergmehl von der Grenzen Lapplands in Schweden Bericht über die zur Bekanntmachung geeigneten. *Abh. K. Preuss. Akad. Wiss., Phys.-Math. Kl* **1837**, 43–45.

Ehrenberg, C.G. *Die Infusionsthierchen als vollkommene Organismen. Ein Blick in das tiefere organische Leben de Natur.*; erlag von Leopold Voss, Leipzig.: Leipzig, Germany, 1838; pp. 1–xvii, 1–548, pls. 1–64. https://doi.org/10.5962/bhl.title.58475

Ehrenberg, C.G. Mittheilungen über 2 neue asiatische Lager fossiler Infusorien-Erden aus dem russischen Trans-Kaukasien (Grusien) und Sibirien Bericht über die zur Bekanntmachung geeigneten. *Abh. K. Preuss. Akad. Wiss., Phys.-Math. Kl* **1843**, 43–49.

Foged, N. (1981). Diatoms in Alaska. In: *Biliotheca Phycologica*, Band 53.; J. Cramer Publishing Co.: Vaduz, Liechtenstein, 1981; pp. 1–317.

Foged, N. (1982). Diatoms in Bornholm, Denmark. *Bibliotheca Phycologica* 59: 1-175.

Frenguelli, J. (1924). Resultados de la Primera Expedición a Tierra del Fuego (1921) - Diatomeas de Tierra de Fuego. *Anales de la Sociedad Científica Argentina* 98: 5-63, 13 pl.

Gregory, W. Notice of the new forms and varieties of known forms occurring in the diatomaceous earth of Mull; with remarks on the classification of the Diatomaceae. *Quarterly Journal of Microscopical Science,* **1854***, 2*: 90–100, pl. IV.

Gregory, W. (1856). Notice of some new species of British fresh-water Diatomaceae. *Quarterly Journal of Microscopical Science, New Series* 4: 1-14, 1 pl.

Gregory, W. (1857). On the post-Tertiary diatomaceous sand of Glenshira. Part II. Containing an account of a number of additional undescribed species. *Transactions of the Microscopical Society of London* 5: 67-88, pl. 1.

Grunow, A. (1862). Die österreichischen Diatomaceen nebst Anschluss einiger neuen Arten von andern Lokalitäten und einer kritischen □ebersicht der bisher bekannten Gattungen und Arten. *Verhandlungen der kaiserlich-königlichen zoologisch-botanischen Gesellschaft in Wien* 12: 315-472 [Abt 1], 545-588 [Abt. 2], 7 pls.

Grunow, A. (1877). New diatoms from Honduras, with notes by F. Kitton. *Monthly Microscopical Journal, London* 18: 165-186, pls 193-196.

Hassall, A.H. *A history of the British Freshwater Algae (including descriptions of the Diatomaceae and Desmidiaceae) with upwards of one hundred Plates. I. Text*. Taylor, Walton, and Maberly: London, England, 1845; pp. 1–462.

Hustedt, F. (1930). Bacillariophyta (Diatomeae) Zweite Auflage. In: *Die Süsswasser-Flora Mitteleuropas. Heft 10*. (Pascher, A. Eds), pp. [i]-vii, [1]-466. Jena: Verlag von Gustav Fischer.

Hustedt, F. Süsswasser-Diatomeen. In *Exploration du Parc National Albert, Mission H. Damas (1935-1936)*.; Institut des Parcs Nationaux du Congo Belge: Brussels, Belgium, 1949; Vol. 8, pp. 1–199.

Hustedt, F. (1937). Systematische und ökologische □ntersuchungen über die Diatomeen-Flora von Java, Bali und Sumatra nach dem Material der Deutschen Limnologischen Sunda-Expedition. *Archiv für Hydrobiologie (Supplement)* 15: 131-177, pls 9-12.

Hustedt, F. Die Kieselalgen Deutschlands, Osterreichs und der Schweiz unter Berucksichtigung der ubrigen Lander Europas sowie der angrenzenden Meeresgebiete. In *Dr. L. Rabenhorst's Kryptogamen-Flora von Deutschland, Osterreich und der Schweiz,* 3. Teil. Lieferung 4.; Akademische Verlagsgesellschaft Geest & Portig KG: Leipzig, Germany, 1927–1966; Band 7, pp. 557–816 mit Figuren 1592–1788.

Hustedt, F. (1943). Die Diatomeenflora einiger Hochgebirgsseen der Landschaft Davos in den schweizer Alpen. *Internationale Revue der gesamten Hydrobiologie und Hydrographie* 43: 124-197, 225-280.

Hustedt, F. (1957). Die Diatomeenflora des Fluß-systems der Weser im Gebiet der Hansestadt Bremen. *Abhandlungen der Naturwissenschaftlichen Verein zu Bremen* 34(3): 181-440, 1 pl.

Hustedt, F. (1959). Die Diatomeenflora des Salzlackengebietes im östereichischen Burgenland. *Österreichchischen Akademie der Wissenschaften, Mathematische und Naturwissenschafliche, Kl. Abt. 1,* 168(4/5): 387-452, 1 pl.

Jüttner, I.; Kociolek, J.P.; Gurung, S.; Gurung, A.; Sharma, C.M.; Levkov, Z.; Williams, D.M.; Ector, L. The genus *Gomphonema* (Bacillariophyta) in Rara Lake, Nepal: taxonomy, morphology, habitat distribution and description of five new species, and a new record for *Gomphoneis qii*. *Diatom Res.* **2018**, *33*(3): 283–320. https://doi.org/10.1080/0269249X.2018.1528182

Kobayasi, H.; Nagumo, T. Examination of the type materials of *Navicula subtilissima* Cleve (Bacillariophyceae). *Bot. Mag. Tokyo* **1988**, *101*, 239–253. https://doi.org/10.1007/BF02488602

Kociolek, J.P. & Stoermer, E.F. (1987). □ltrastructure of *Cymbella sinuata* and its allies (Bacillariophyceae), and their transfer to *Reimeria*, gen. nov. *Systematic Botany* 12(4): 451-459, 25 figs.

Kociolek, J.P.; Blanco, S.; Coste, M.; Ector, L.; Liu, Y.; Karthick, B.; Kulikovskiy, M.; Lundholm, N.; Ludwig, T.; Potapova, M.; Rimet, F.; Sabbe, K.; Sala, S.; Sar, E.; Taylor, J.; Van de Vijver, B.; Wetzel, C.E.; Williams, D.M.; Witkowski, A.; Witkowski, J. (2021). DiatomBase. *Eunotia groenlandica* (Grunow) Norpel-Schempp & Lange-Bertalot in Lange-Bertalot & Metzeltin, 1996. Accessed at: http://diatombase.org/aphia.php?p=taxdetails&id=622039 on 2023-03-04

Krammer, K. & Lange-Bertalot, H. (1985). Naviculaceae Neue und wenig bekannte Taxa, neue Kombinationen und Synonyme sowie Bemerkungen zu einigen Gattungen. *Bibliotheca Diatomologica* 9: [1]-230, pls 1-43.

Krammer, K. Valve morphology and taxonomy in the genus *Stenopterobia* (Bacillariophyceae). *Brit. Phycol. J.* **1989**, *24*(3), 237–243. https://doi.org/10.1080/00071618900650261

Krammer, K. (1992). *Pinnularia*. Eine Monographie der europäischen Taxa. *Bibliotheca Diatomologica* 26: 1-353, pls 1-76.

Krammer, K. *Die cymbelloiden Diatomeen: Eine Monographie der weltweit bekannten Taxa. Teil 1. Allgemeines und Encyonema* Part. Band 36. In *Bibliotheca Diatomologica,* Lange-Bertalot, H.; Kociolek, P. Eds.; J. Cramer: Berlin, Germany, 1997; pp. 1–382.

Krammer, K. & Lange-Bertalot, H. (1985). Naviculaceae Neue und wenig bekannte Taxa, neue Kombinationen und Synonyme sowie Bemerkungen zu einigen Gattungen. *Bibliotheca Diatomologica* 9: [1]-230, pls 1-43.

Krammer, K.; Lange-Bertalot, H. *Bacillariophyceae, teil 2. Epithemiaceae, Bacillariophyceae, Surirellaceae.;* Spektrum Akademischer Verlag: Heidelberg, Germany, 1988; pp. 1–612.

Krammer, K.; Lange-Bertalot, H. *Bacillariophyceae, teil 3. Centrales, Fragilariaceae, Eunotiaceae, Achnanthaceae*.; Spektrum Akademischer Verlag: Heidelberg, Germany, 1991; pp. 1–576.

Krammer, K.; Lange-Bertalot, H. *Bacillariophyceae, teil 4. Achnanthaceae, kritische erganzungen zu Navicula (lineolate) und Gomphonema.*; Spektrum Akademischer Verlag: Heidelberg, Germany, 1991; pp. 1–437.

Krammer, K. *Diatoms of Europe: The genus Pinnularia,* Vol. 1; ARG Gantner Verlag KG: Ruggell, Liechtenstein, 2000; pp. 1–703.

Krasske, G. (1929). Beitrage zur Kenntnis der Diatomeenflora Sachsens. *Botanisches Archiv* 27(3/4): 348-380, 1 pl.

Krasske, G. (1932). Beiträge zur Kenntnis der Diatomeenflora der Alpen. *Hedwigia* 72(3): 92-135, pls 1, 2.

Krasske, G. Die Diatomeenflora der Moosrasen des Wilhelmshoher Parkes. In *Festschrift des Vereins fur Naturkunde zu Kassel zum hundertjahrigen Bestehen*. Druck von L. Döll: Kassel, Germany, 1936; pp. 151–64.

Kulikovskiy, M., Lange-Bertalot, H., Genkal, S. & Witkowski, A. (2010). *Eunotia* (Bacillariophyta) in the Holarctic: new species from the Russian Arctic. *Polish Botanical Journal* 55(1): 93-106, 6 figs.

Kulikovskiy, M.S., Lange-Bertalot, H. & Kuznetsova, I.V. (2015). Lake Baikal: hotspot of endemic diatoms II. *Iconographia Diatomologica* 26: [1]-656.

Kützing, F.T. *Synopsis diatomearum oder Versuch einer systematischen Zusammenstellung der Diatomeen,* Linnaea, Bd. 8, Helf 5; Schwetschke: Berlin, Germany, 1834; pp. 529–620, + pls XIII–XIX [79 figs]. https://doi.org/10.5962/bhl.title.65634

Kützing, F.T. *Die Kieselschaligen. Bacillarien oder Diatomeen.* Zu finden bei W. Köhne: Nordhausen, Germany, 1844; pp. 1–152. https://doi.org/10.5962/bhl.title.64360

Kützing, F.T. *Species algarum*. F.A. Brockhaus: Leipzig, Germany, 1849; pp. i–vi, 1–922.

Lange-Bertalot, H. (1980). Ein beitrag zur revision der Gattungen *Rhoicosphenia* Grun., *Gomphonema* C. Ag., *Gomphoneis* Cl.. *Botaniska Notiser* 133: 585-594.

Lange-Bertalot, H. (1980). New species, combinations and synonyms in the genus *Nitzschia*. *Bacillaria* 3: 41-77.

Lange-Bertalot, H. (1993). 85 neue Taxa und über 100 weitere neu definierte Taxa ergänzend zur Süsswasserflora von Mitteleuropa, Vol. 2/1-4. 85 New Taxa and much more than 100

taxonomic clarifications supplementary to SüBwasserflora von Mitteleuropa Vol. 2/ 1-4. *Bibliotheca Diatomologica* 27: 1-454, incl. 1427 figs in 134 pls.

Lange-Bertalot, H. *Kobayasiella* nom. nov. ein neuer Gattungsname für *Kobayasia* Lange-Bertalot 1996. In, *Iconographia Diatomologica. Annotated Diatom Micrographs,* Lange-Bertalot, H. Ed.; Koeltz Scientific Books: Königstein, Germany, 1999; Vol. 6, pp. 272–275.

Lange-Bertalot, H.; Genkal, S.I. Diatoms from Siberia I: Islands in the Arctic Ocean (Yugorsky–Shar Strait). Vol 6; In *Iconographia Diatomologica, Annotated Diatom Micrographs.*; Lange-Bertalot, H. Ed.; A.R.G. Gantner Verlag K.G.: Vaduz, Liechtenstein, 1999; pp. 1–271.

Lange-Bertalot, H.; Krammer, K. *Diatoms of Europe: Navicula sensu stricto, 10 genera separated from Navicula sensu lato. Frustulia* Vol. 2; ARG Gantner Verlag KG: Ruggell, Liechtenstein, 2001; pp. 1–526.

Lange-Bertalot, H., Cavacini, P., Tagliaventi, N. & Alfinito, S. (2003). Diatoms of Sardinia. Rare and 76 new species in rock pools and other ephemeral waters. *Iconographia Diatomologica* 12: 1-438, 1369 figs in 137 pls

Lange-Bertalot, H., Bak, M., Witkowski, A., & Tagliaventi, N. Diatoms of Europe: Eunotia and some related genera Vol. 6; Gantner Verlag KG: Ruggell, Liechtenstein, 2011; pp. 1–747.

Lange-Bertalot, H.; Hofmann, G.; Werum, M.; Cantonati, M.; Kelly, M. G. Freshwater benthic diatoms of Central Europe: over 800 common species used in ecological assessment, Vol. 942.; Koeltz Botanical Books: Schmitten-Oberreifenberg, Hessen,Germany, 2017; pp. 1–908.

Lewis, F.W. On some new and singular intermediate forms of Diatomaceae. *Proc. Acad. Nat. Sci. Philadelphia* **1864**, *15*, 336–346.

Lyngbye, H.C. *Tentamen Hydrophytologiae Danicae Continens omnia Hydrophyta Cryptogama Daniae, Holsatiae, Faeroae, Islandiae, Groenlandiae hucusque cognita, Systematice Disposita, Descripta et iconibus illustrata, Adjectis Simul Speciebus Norvegicis*. Fb&c Limited: Copenhagen, Denmark, 1819; pp. 1–248.

Migula, W. *Kryptogamen-Flora von Deutschland, Deutsch-Österreich und der Schweiz. Band II. Algen. 1. Teil. Cyanophyceae, Diatomaceae, Chlorophyceae*. H. Bergmühler: Berlin, Germany, 1905; pp. 1–208.

Metzeltin, D.; Lange-Bertalot, H. Tropical Diatoms of South America II. Special remarks on biogeography disjunction. In *Iconographia Diatomologica*; A.R.G. Gantner: Ruggell, Liechtenstein, 2007; Vol. 18, pp. 1–877, incl. 296 pl.

Mölder, K. (1951). Beitrage zur Kenntnis der rezenten Diatomeenflora Ostkareliens. *Annales Botanici Societatis Zoologicae Botanicae Fennicae, Vanamo, Helsinki* 25(1): 1-35.

Morales, E. & Manoylov, K.M. (2006). Morphological studies on selected taxa in the genus *Staurosirella* Williams et Round (Bacillariophyceae) from rivers in North America. *Diatom Research* 21(2): 343-364, 121 fig., 1 table.

Morales, E.A. & Vis, M.L. (2007). Epilithic diatoms (Bacillariophyceae) from cloud forest and alpine streams in Bolivia, South America. *Proceedings of the Academy of Natural Sciences of Philadelphia* 156: 123-155, 292 figs.

Nakov, T.; Guillory, W.; Julius, M.; Theriot, E.; Alverson, A. Towards a phylogenetic classification of species belonging to the diatom genus *Cyclotella* (Bacillariophyceae): Transfer of species formerly placed in *Puncticulata, Handmannia, Pliocaenicus* and *Cyclotella* to the genus *Lindavia*. *Phytotaxa* **2015**, *217*(3), 249–264. https://doi.org/10.11646/phytotaxa.217.3.2

Patrick, R.M. & Freese, L.R. (1961). Diatoms (Bacillariophyceae) from Northern Alaska. *Proceedings of the Academy of Natural Sciences of Philadelphia* 112(6): 129-293, 4 pls.

Østrup, E. Ferskvands-Diatoméer fra Øst Grønland. *Meddelelser om Grønland,* **1897**, *15*, 251–290.

Rabenhorst, L. (1848-1860). *Die Algen Sachsens*. Resp. Mittel-Europa's Gesammelt und herausgegeben von Dr. L. Rabenhorst, Dec. 1-100. No. 1-1000. [Exsiccata, issued at various dates]. . Dresden.

Rabenhorst, L. *Die Süsswasser-Diatomaceen (Bacillarien) für Freunde der Mikroskopie*. Eduard Kummer: Leipzig, Germany, 1853; pp. 1–72, 9 pls.

Rabenhorst, L. (1864). Flora europaea algarum aquae dulcis et submarinae. Sectio I. Algas diatomaceas complectens, cum figuris generum omnium xylographice impressis. pp. 1-359. Lipsiae [Leipzig]: Apud Eduardum Kummerum.

Reichardt, E. & Lange-Bertalot, H. (1991). Taxonomische Revision des Artencomplexes um *Gomphonema angustum—G. dichotomum—G. intricatum—G. vibrio* und ahnliche Taxa (Bacillariophyceae). *Nova Hedwigia* 53(3-4): 519-544.

Round, F. E.; Crawford, R. M.; Mann, D. G. *Diatoms: biology and morphology of the genera.* Cambridge university press: Cambridge, England, 1990; pp. 1–747.

Round, F.E. & Bukhtiyarova, L. (1996). Four new genera based on *Achnanthes (Achnanthidium)* together with a re-definition of *Achnanthidium*. *Diatom Research* 11(2): 345-361.

Rumrich, □., Lange-Bertalot, H. & Rumrich, M. (2000). Diatomeen der Anden von Venezuela bis Patagonien/Feuerland und zwei weitere Beiträge. Diatoms of the Andes from Venezuela to Patagonia/Tierra del Fuego and two additional contributions. *Iconographia Diatomologica* 9: 1-673, incl. 197 pls.

Schmidt, A.[W.F.] (1913). *Atlas der Diatomaceen-kunde* Series VI: Heft [72]. pp. pls 285-288[F. Hustedt]. Leipzig: O.R. Reisland.

Schumann, J. (1867). Preussische Diatomeen. *Schriften der koniglichen physikalisch-okonomischen Gesellschaft zu Konigsberg* 8: 37-68, pls 1-3.

Shi Zhixin [Shi, Z.X.] *et al.* (2004). *Flora algarum sinicarum aquae dulcis. Tomus XII Bacillariophyta Gomphonemataceae*. pp. i-xviii, 1-147, incl. XXXVII pl. Beijing: Science Press.

Skvortsov, B.V. (1938). Diatoms from a peaty bog in Lianchicho River Valley, Eastern Siberia. *Philippine Journal of Science. Section C* 66(2): 161-182, 3 pls.

Smith, W. (1856). *A synopsis of the British Diatomaceae*; with remarks on their structure, functions and distribution; and instructions for collecting and preserving specimens. The plates by Tuffen West. In two volumes. Vol. II. pp. [i]-xxix, 1-107, pls 32-60, 61-62, A-E. London: John van Voorst, Paternoster Row.

Sovereign, H.E. (1960). The diatoms of Crater Lake, Oregon Supplementary note. *Transactions of the American Microscopical Society* 79(3): 345 only, no fig.

Spaulding et al. 2021. Diatoms.org: supporting taxonomists, connecting communities. Diatom Research 36(4): 291-304. doi:10.1080/0269249X.2021.2006790

Thomas, E.W. & Kociolek, J.P. (2015). Taxonomy of three new *Rhoicosphenia* (Bacillariophyta) species from California, □SA. *Phytotaxa* 204(1): 1-21, 110 fig., 3 tables.

Thwaites, G.H.K. Further observations on the Diatomaceae with descriptions of new genera and species. *Ann. Mag. nat. Hist.* **1848**, *2*(1), 161–172. https://doi.org/10.1080/03745485809496091

Van Heurck, H. *A treatise on the Diatomaceae*. Translated by W.E. Baxter; William Wesley & Son: London, 1896; pp. 1–558, pls 1–35.

Van Heurck, H. (1881). *Synopsis des Diatomées de Belgique* Atlas. pp. pls XXXI-LXXVII [31-77]. Anvers: Ducaju et Cie.

Van Heurck, H. (1896). *A treatise on the Diatomaceae*. Translated by W.E. Baxter. pp. 1-558, pls 1-35. London: William Wesley & Son.

Wallace, J.H. (1960). New and variable diatoms. *Notulae Naturae (Philadelphia)* 331: 1-8, 2 pls.

Werum, M. & Lange-Bertalot, H. (2004). Diatoms in springs from Central Europe and elsewhere under the influence of hydrologeology and anthropogenic impacts. *Iconographia Diatomologica* 13: 3-417, pls 1-105.

Williams, D.M. & Round, F.E. (1988 '1987'). Revision of the genus *Fragilaria*. *Diatom Research* 2: 267-288, 58 figs.

Williams, D.M.; Round, F.E. (1988) *Fragilariforma, nom. nov*., a new generic name for *Neofragilaria* Williams Round. *Diatom Res.* **1988**, *3*(2), 265–267. https://doi.org/10.1080/0269249X.1988.9705039

Witkowski, A., Lange-Bertalot, H. & Metzeltin, D. (2000). Diatom flora of marine coasts I. *Iconographia Diatomologica* 7: 1-925, incl. 219 pls with 4504 figs.
